# miR-199a functions downstream of MeCP2 in neurons of *MECP2* duplication syndrome models

**DOI:** 10.1016/j.isci.2025.113789

**Published:** 2025-10-16

**Authors:** Yuichi Akaba, Satoru Takahashi, Shota Adachi, Masatoshi Nishimura, Keiichiro Suzuki, Hideyuki Nakashima, Kinichi Nakashima, Ryutaro Kira, Pin Fee Chong, Yasunari Sakai, Yohei Hayashi, Itaru Kushima, Daisuke Mori, Yuko Arioka, Hiroki Okumura, Atsuo Nakayama, Seiji Mizuno, Toshiyuki Yamamoto, Fumitaka Osakada, Norio Ozaki, Keita Tsujimura

**Affiliations:** 1 Department of Pediatrics, Asahikawa Medical University, Asahikawa, Japan; 2 Research Unit for Developmental Disorders, Institute for Advanced Research, Nagoya University, Nagoya, Japan; 3 Group of Brain Function and Development, Nagoya University Neuroscience Institute of the Graduate School of Science, Nagoya, Japan; 4 Laboratory of Cellular Pharmacology, Graduate School of Pharmaceutical Sciences, Nagoya University, Nagoya, Japan; 5 Institute for Advanced Co-Creation Studies, The University of Osaka, Toyonaka, Japan; 6 Graduate School of Engineering Science, The University of Osaka, Toyonaka, Japan; 7 Graduate School of Frontier Bioscience, The University of Osaka, Suita, Japan; 8 Department of Stem Cell Biology and Medicine, Graduate School of Medical Sciences, Kyushu University, Fukuoka, Japan; 9 Department of Pediatric Neurology, Fukuoka Children’s Hospital, Fukuoka, Japan; 10 Department of Pediatrics, Kyushu University, Fukuoka, Japan; 11 Research and Development Center, CiRA Foundation, Osaka, Japan; 12 Medical Genomics Center, Nagoya University Hospital, Nagoya, Japan; 13 Department of Psychiatry, Graduate School of Medicine, Nagoya University, Nagoya, Japan; 14 Brain and Mind Research Center, Nagoya University, Nagoya, Japan; 15 Pathophysiology of Mental Disorders, Graduate School of Medicine, Nagoya University, Nagoya, Japan; 16 Center for Advanced Medicine and Clinical Research, Nagoya University Hospital, Nagoya, Japan; 17 Research Unit for Developmental Disorders, Institute for Advanced Research, Nagoya University, Nagoya, Japan; 18 Department of Hospital Pharmacy, Nagoya University Hospital, Nagoya, Japan; 19 Department of Cellular Pathology, Institute for Developmental Research, Aichi Developmental Disability Center, Kasugai, Japan; 20 Department of Neurochemistry, Graduate School of Medicine, Nagoya University, Nagoya, Japan; 21 Department of Pediatrics, Central Hospital, Aichi Developmental Disability Center, Kasugai, Japan; 22 Institute of Medical Genetics, Tokyo Women’s Medical University, Tokyo, Japan; 23 Division of Gene Medicine, Graduate School of Medicine, Tokyo Women’s Medical University, Tokyo, Japan; 24 Laboratory of Neural Information Processing, Institute for Advanced Research, Nagoya University, Nagoya, Japan; 25 PRESTO/CREST, Japan Science and Technology Agency, Saitama, Japan; 26 Rett Syndrome Organization Japan, Hirakata, Japan

**Keywords:** Natural sciences, Biological sciences, Neuroscience

## Abstract

Duplication of the methyl-CpG-binding protein 2 (*MECP2*) gene causes *MECP2* duplication syndrome (MDS), a severe neurodevelopmental disorder with an unclear pathology. We previously showed that MeCP2 promotes the processing of specific microRNAs (miRNAs), including miR-199a, to regulate neuronal functions. Here, we demonstrate that neurons derived from MDS model mice and patient-induced pluripotent stem cells (iPSCs) exhibit morphological abnormalities, such as abnormal dendrite outgrowth, enlarged soma size, increased glutamatergic synapse density, and hyperactivation of the mechanistic target of rapamycin (mTOR) signaling. MeCP2 overexpression increased miR-199a production in both models. Blocking miR-199a-5p improved soma size and mTOR activity, while inhibiting miR-199a-3p normalized dendritic outgrowth. Crossing MDS model mice with *miR-199a-2* knockout mice ameliorated synaptic and mTOR abnormalities. Human MDS cortical organoids exhibited reduced neuronal activity, which was reversed by suppressing miR-199a-5p. These findings identify miR-199a as a key downstream mediator of MeCP2 in MDS, providing new insights into its molecular pathology.

## Highlights

• miR-199a mediates MeCP2-induced abnormal neuronal morphology in MDS models
• Inhibition of miR-199a rescues dendritic and soma abnormalities in MDS neurons
• miR-199a inhibition reduces mTOR activity in MDS models *in vitro* and *in vivo*

## Graphical abstract

**Figure:**
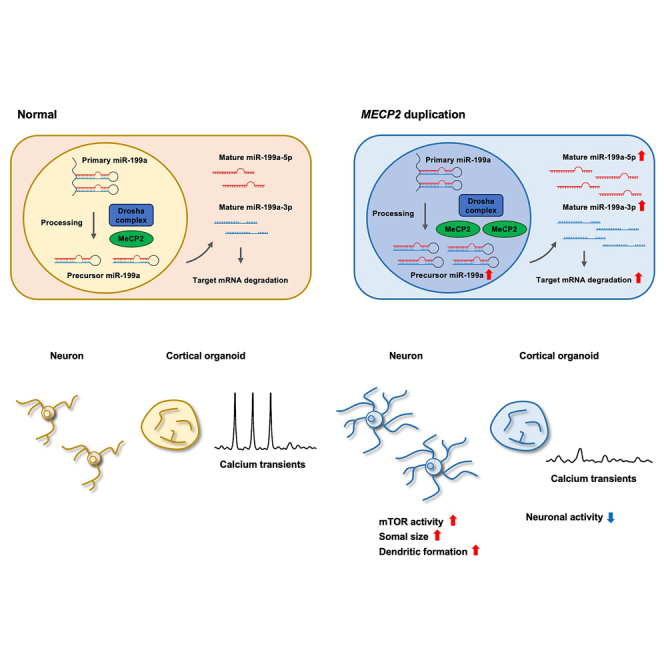

## Introduction

Methyl-CpG binding protein 2 (MeCP2) is a multifunctional epigenetic modulator involved in the regulation of gene expression and is required for proper brain development.^1^^,^^2^ MeCP2, encoded by the *MECP2* gene located on Xq28, is highly expressed in the brain, where its abundance of MeCP2 increases during neuronal maturation and synaptogenesis.^3^^,^^4^ MeCP2 was initially proposed to act as a transcriptional repressor by binding to the co-repressor Sin3A and recruiting the histone deacetylase (HDAC) complex.^5^ However, subsequent studies have revealed additional epigenetic roles for MeCP2, including transcriptional activation that varies with the molecular context, regulation of mRNA splicing, and microRNA (miRNA) processing.^1^^,^^6^^,^^7^^,^^8^ Loss-of-function variants in the *MECP2* gene in females lead to Rett syndrome (RTT; OMIM 312750), a severe neurodevelopmental disorder characterized by normal development until 6–18 months of age, followed by rapid regression. This regression results in the loss of acquired purposeful hand skills and acquired spoken language, along with the emergence of autism, seizures, and stereotypic hand movements.^9^^,^^10^ Loss-of-function variants in the *MECP2* gene in males have been considered to cause fetal lethality or early postnatal death because no wild-type (WT) allele is expressed, unlike in females. However, recent reports describe surviving males with hypomorphic variants, somatic mosaicism, or an additional X chromosome.^11^ In contrast, duplication or extra copies of the *MECP2* gene cause a distinct disorder, *MECP2* duplication syndrome (MDS; OMIM 300283). The common phenotypes of MDS include early onset hypotonia, severe intellectual disability, progressive spasticity, recurrent infections, autism, and seizures.^12^^,^^13^^,^^14^ The MDS phenotype appears mainly in males, while in females, a duplication of one of the two copies of the *MECP2* gene typically does not cause the disorder. However, it can be associated with neurological symptoms such as anxiety, depression, and features of autism spectrum disorder (ASD). Its severity ranges from anxiety and mild intellectual disability to a severe phenotype similar to that observed in males, depending on the consequence of random X chromosome inactivation.^15^ Although both RTT and MDS are caused by alterations in MeCP2 dosage and share overlapping clinical features such as intellectual disability, epilepsy, and motor dysfunction, they exhibit distinct clinical courses. RTT is characterized by a period of normal early development followed by regression, whereas MDS typically presents with symptoms from early infancy.^16^^,^^17^

*MECP2* transgenic mouse lines, generated using a large genomic clone containing the entire human *MECP2* locus, have been utilized as *in vivo* models for MDS.^18^ Notably, transgenic lines that expressed human *MECP2* exhibited progressive neurological phenotypes with varied severity corresponding to MeCP2 protein levels, where mice from higher-expressing lines manifested more severe abnormalities than did mice from lower-expressing lines. Transgenic mice (Tg1) expressing MeCP2 at twice the WT levels were found to develop age-worsening seizures, become hypoactive, and have a 30% mortality rate by 1 year of age.^18^ Recent research on mice has demonstrated encouraging results showing that restoring MeCP2 levels to normal in symptomatic mice can reverse their phenotype.^19^ Ablation of one allele in adult male mice carrying two functional MeCP2 alleles, with one allele being a human *MECP2* transgene and the other being a conditional mouse *Mecp2* that can be deleted by tamoxifen-inducible Cre recombination, reversed neurological abnormalities within 6–7 weeks.^19^ A challenge with this therapeutic approach is the need to maintain MeCP2 levels within an appropriate range, as reducing its expression below normal levels would likely cause severe neurological deficits. These findings support the concept that alterations in MeCP2 levels are not only causal but also therapeutically targetable, reinforcing the value of these models for translational research.

miRNAs are endogenous small non-coding RNAs comprising approximately 22 nucleotides that can negatively regulate gene expression at the post-transcriptional level. Additionally, they play important roles in many biological processes, such as cell proliferation, differentiation, and development.^20^ miRNAs interact with their complementary sequence on the 3′ untranslated region (UTR) of mRNAs to inhibit protein translation or promote degradation of target mRNAs.^21^ A single miRNA can bind to hundreds of target sites in the 3′UTR of mRNAs, thus regulating a large variety of molecular pathways involved in the pathophysiology of various neurological disorders such as schizophrenia, major depressive disorder, and ASD.^22^^,^^23^^,^^24^ Recent studies have shown that MeCP2 interacts with the miRNA machinery.^25^^,^^26^^,^^27^ Neuronal activity induces brain-derived neurotrophic factor (BDNF) expression, which is controlled by MeCP2.^28^ miR-132 is induced by BDNF and negatively regulates *Mecp2* mRNA by binding to its 3′UTR.^25^ The loss of MeCP2 reduces BDNF and miR-132 levels.^27^^,^^28^ Since BDNF and miR-132 promote dendritic growth and neuronal morphogenesis,^29^ the BDNF/miR-132/MeCP2 feedback loop is thought to play a critical role in activity-dependent neuronal development. miR-483-5p, an intragenic miRNA of the imprinted gene insulin-like growth factor 2, plays a crucial role in regulating MeCP2 levels in human fetal brains.^26^ Expression of miR-483-5p in hippocampal neurons rescues the abnormal dendritic spine phenotype induced by excess human MeCP2.^26^ Recently, we reported that MeCP2 promotes the processing of miR-199a as a component of Drosha, a microprocessor complex responsible for cleaving primary miRNA transcripts to produce miRNA precursors.^8^ We also found that *miR-199a-2* knockout (KO) mice recapitulated many of the phenotypes observed in the *Mecp2*-KO mouse model of RTT.^8^ Additionally, we reported that the MeCP2/miR-199a axis inhibits bone morphogenetic protein (BMP)-Smad signaling by targeting *Smad* family member 1 (*Smad1*) and regulates neural stem/precursor cell differentiation.^27^ Although the MeCP2/miR-199a pathway has been implicated in neurodevelopmental processes, its relevance to the pathophysiology of MDS remains unclear. As miR-199a is directly regulated by MeCP2, investigating its role in MDS may provide novel insights into disease mechanisms and identify a potential therapeutic target.

## Results

### MDS model neurons exhibit abnormal morphology and mTOR hyperactivation

**Figure 1:**
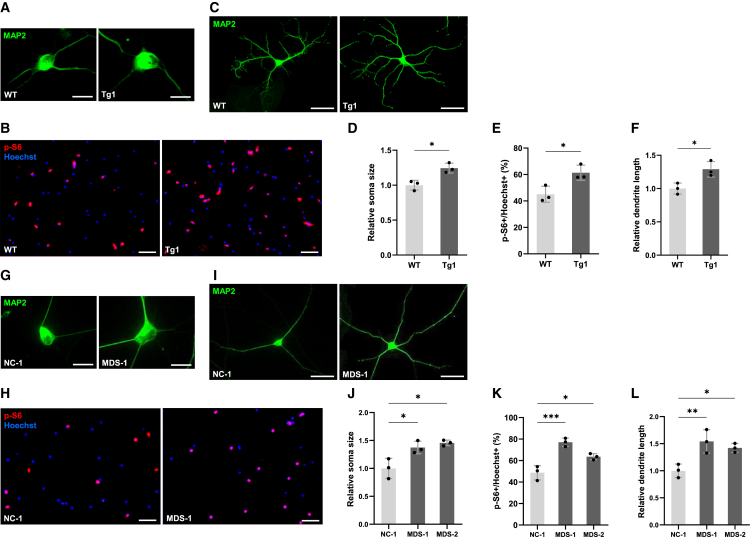
MDS model neurons show neuronal overgrowth and increased mTOR activity (A and C) Representative images of mouse hippocampal neurons stained with anti-MAP2 (green) antibody at 7 DIV. Scale bars: 20 μm (A), 50 μm (C). (B) Representative images of mouse hippocampal neurons stained with anti-p-S6 (red) antibody and Hoechst 33258 (blue) at 7 DIV. Scale bars: 100 μm. (D–F) Quantification of neuronal soma size (D), mTOR activity (E), and total dendrite length (F) at 7 DIV. Data are presented as mean ± SD from three independent experiments (*n* = 3). In all experiments, at least 52 neurons were analyzed per condition. The average relative soma size was 1.00 ± 0.07 in WT and 1.24 ± 0.07 in Tg1 neurons, and relative dendrite length was 1.00 ± 0.08 in WT and 1.29 ± 0.12 in Tg1 neurons. p-S6-positive cells accounted for 45.0% ± 6.1% of Hoechst-stained cells in WT and 61.3% ± 5.7% in Tg1 neurons. Statistical significance was determined using Student’s t test (∗*p* < 0.05). (G and I) Representative images of human iPSC-derived neurons stained with anti-MAP2 (green) antibody at 7 DIV. Scale bars: 20 μm (G), 50 μm (I). (H) Representative images of human iPSC-derived neurons stained with anti-p-S6 (red) antibody and Hoechst 33258 (blue) at 7 DIV. Scale bars: 100 μm. (J–L) Quantification of neuronal soma size (J), mTOR activity (K), and total dendrite length (L) was performed at 7 DIV using iPSC-derived neurons from three lines: NC-1 (201B7), MDS-1 (AC1140206AS), and MDS-2 (HiPS-AC8783). Data are presented as mean ± SD from three independent experiments (*n* = 3). In all experiments, at least 51 neurons were analyzed per condition. The average relative soma size was 1.00 ± 0.18 in NC-1, 1.37 ± 0.11 in MDS-1, and 1.45 ± 0.05 in MDS-2. Relative dendrite length was 1.00 ± 0.12 in NC-1, 1.54 ± 0.21 in MDS-1, and 1.42 ± 0.08 in MDS-2. p-S6-positive cells accounted for 48.7% ± 6.6% of Hoechst-stained cells in NC-1, 77.1% ± 3.9% in MDS-1, and 63.6% ± 2.8% in MDS-2. Statistical significance was determined using Tukey’s multiple comparison test (∗*p* < 0.05, ∗∗*p* < 0.01, ∗∗∗*p* < 0.001).

Previous studies have demonstrated that the loss of MeCP2 function inhibits the activation of mechanistic target of rapamycin (mTOR) signaling and reduces neuronal soma growth.^8^^,^^30^ Thus, we first examined the changes in the soma size of hippocampal neurons derived from MDS model (Tg1) mice and found that the soma size was larger in MDS neurons than in WT neurons (Figures 1A and 1D). To investigate mTOR signaling activity in MDS neurons, we further performed immunofluorescence staining with an antibody for the phosphorylation of ribosomal protein S6 (p-S6, Ser235/236), a reliable marker of mTOR pathway (especially for mTORC1 pathway) activation, at 7 days *in vitro* (DIV). Immunocytochemistry analysis revealed that the number of p-S6-positive cells was markedly larger in hippocampal neurons prepared from Tg1 mice than that from WT mice (Figures 1B and 1E). Proper dendritic development is crucial for the normal functioning of the central nervous system (CNS), and its dysregulation is implicated in the pathophysiology of neurodevelopmental disorders.^31^ To explore the morphological changes in the dendrites of MDS neurons, we evaluated dendritic outgrowth in cultured primary hippocampal neurons prepared from Tg1 mice by performing immunocytochemistry using an anti-MAP2 antibody at 7 DIV. We found that the total dendrite length was significantly greater in hippocampal neurons derived from Tg1 mice than those prepared from WT mice (Figures 1C and 1F). To validate these animal model findings in a human context, we generated patient-induced pluripotent stem cells (iPSCs) (AC1140206AS and HiPS-AC8783) from two patients with MDS and evaluated the phenotypes of iPSC-derived neurons. Western blot analysis confirmed that MeCP2 protein expression was approximately 2-fold higher in MDS iPSC-derived neurons compared with those from normal control (NC) individuals, consistent with *MECP2* gene duplication (Figures S1A and S1B). At 7 days post differentiation, neurons were immunostained with anti-MAP2 and anti-p-S6 antibodies. iPSC-derived neurons from patients with MDS, like hippocampal neurons of Tg1 mice, exhibited abnormal enhancement of neuronal soma growth, mTOR activity, and dendritic outgrowth compared with NC-derived neurons (Figures 1G–1L). To confirm mTOR pathway activation in MDS iPSC-derived neurons, we performed western blot analysis using antibodies against phosphorylated mTOR (*p*-mTOR, Ser2448) and phosphorylated S6 (p-S6, Ser235/236). Both markers showed significantly increased phosphorylation levels in MDS neurons compared with NC neurons, supporting enhanced mTOR signaling activity in the patient-derived cellular model (Figures S1A, S1C, and S1D). In addition, immunocytochemical analysis of these markers (p-S6/S6 and *p*-mTOR/mTOR) further confirmed the enhanced activation of the mTOR pathway in MDS iPSC-derived neurons (Figures S1E–S1H). To examine whether the morphological changes in MDS iPSC-derived neurons reflect an inherent capacity, we analyzed soma size and dendritic outgrowth at the later timepoint. MDS iPSC-derived neurons continued to exhibit abnormal enhancement of soma growth and dendritic arborization at 14 days post differentiation compared with NC-derived neurons, being similar to the findings at 7 days (Figures S1I–S1L).

### Knockdown of MeCP2 improves abnormal neuronal morphology and mTOR hyperactivation in MDS model neurons

**Figure 2:**
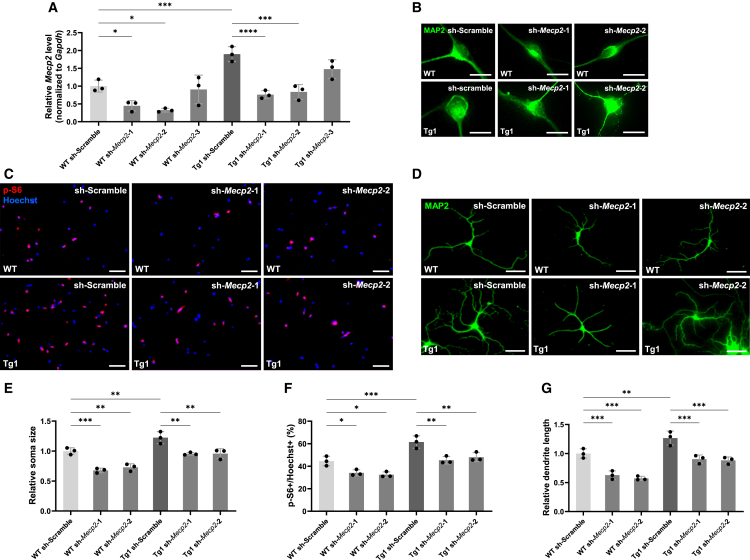
Knockdown of *Mecp2* suppresses neuronal growth and mTOR activity in hippocampal neurons of Tg1 mice (A) Knockdown efficiency of shRNAs was determined by RT-qPCR in WT and Tg1 mouse hippocampal neurons. Total RNA was isolated at 7 DIV after infection with lentiviruses expressing shRNAs. Data are presented as mean ± SD from three independent experiments (*n* = 3). The relative *Mecp2* expression levels (normalized to *Gapdh*) were: WT sh-Scramble, 1.00 ± 0.17; WT sh-*Mecp2*-1, 0.45 ± 0.15; WT sh-*Mecp2*-2, 0.32 ± 0.05; WT sh-*Mecp2*-3, 0.91 ± 0.40; Tg1 sh-Scramble, 1.90 ± 0.21; Tg1 sh-*Mecp2*-1, 0.76 ± 0.11; Tg1 sh-*Mecp2*-2, 0.84 ± 0.21; Tg1 sh-*Mecp2*-3, 1.48 ± 0.26. Statistical significance was determined using Tukey’s multiple comparison test (∗*p* < 0.05, ∗∗∗*p* < 0.001, ∗∗∗∗*p* < 0.0001). (B and D) Representative images of mouse hippocampal neurons stained with anti-MAP2 (green) antibody at 7 DIV after infection with lentiviruses expressing shRNAs. Scale bars: 20 μm (B), 50 μm (D). (C) Representative images of mouse hippocampal neurons stained with anti-p-S6 (red) antibody and Hoechst 33258 (blue) at 7 DIV after infection with lentiviruses expressing shRNAs. Scale bars: 100 μm. (E–G) Quantification of neuronal soma size (E), mTOR activity (F), and total dendrite length (G) at 7 DIV. Data are presented as mean ± SD from three independent experiments (*n* = 3). In all experiments, at least 50 neurons were analyzed per condition. The average relative soma size was 1.00 ± 0.06 in WT sh-Scramble, 0.68 ± 0.04 in WT sh-*Mecp2*-1, 0.73 ± 0.06 in WT sh-*Mecp2*-2, 1.22 ± 0.10 in Tg1 sh-Scramble, 0.95 ± 0.02 in Tg1 sh-*Mecp2*-1, and 0.95 ± 0.06 in Tg1 sh-*Mecp2*-2. Relative dendrite length was 1.00 ± 0.08 in WT sh-Scramble, 0.63 ± 0.07 in WT sh-*Mecp2*-1, 0.57 ± 0.04 in WT sh-*Mecp2*-2, 1.27 ± 0.12 in Tg1 sh-Scramble, 0.90 ± 0.07 in Tg1 sh-*Mecp2*-1, and 0.88 ± 0.06 in Tg1 sh-*Mecp2*-2. p-S6-positive cells accounted for 44.6% ± 4.3% of Hoechst-stained cells in WT sh-Scramble, 34.1% ± 2.7% in WT sh-*Mecp2*-1, 32.5% ± 2.5% in WT sh-*Mecp2*-2, 61.5% ± 5.5% in Tg1 sh-Scramble, 45.4% ± 3.3% in Tg1 sh-*Mecp2*-1, and 48.1% ± 4.0% in Tg1 sh-*Mecp2*-2. Statistical significance was determined using Tukey’s multiple comparison test (∗*p* < 0.05, ∗∗*p* < 0.01, ∗∗∗*p* < 0.001).

**Figure 3:**
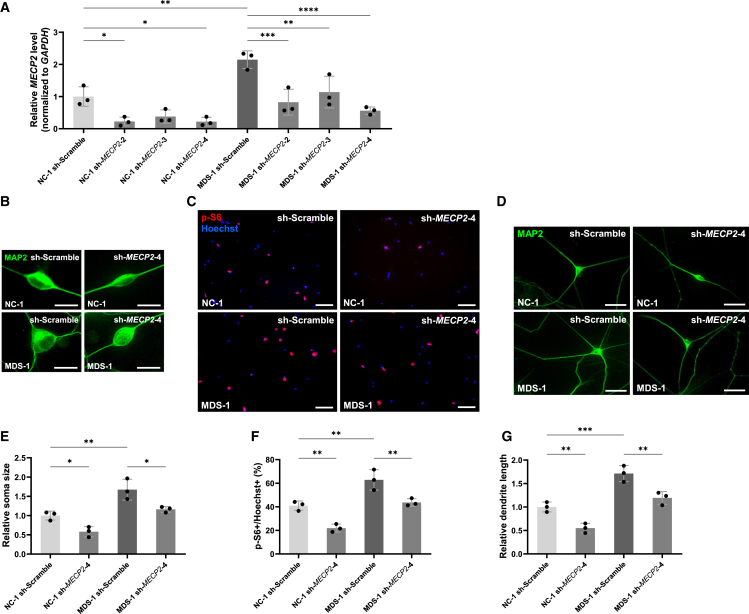
Knockdown of *MECP2* suppresses neuronal growth and mTOR activity in iPSC-derived MDS patient neurons (A) Knockdown efficiency of shRNAs was determined by RT-qPCR in human iPSC-derived neurons. Total RNA was isolated at 7 DIV after infection with lentiviruses expressing shRNAs. Data are presented as mean ± SD from three independent experiments (*n* = 3). The relative *MECP2* expression levels (normalized to *GAPDH*) were: NC-1 sh-Scramble, 1.00 ± 0.30; NC-1 sh-*MECP2*-2, 0.22 ± 0.14; NC-1 sh-*MECP2*-3, 0.38 ± 0.21; NC-1 sh-*MECP2*-4, 0.22 ± 0.13; MDS-1 sh-Scramble, 2.15 ± 0.27; MDS-1 sh-*MECP2*-2, 0.82 ± 0.40; MDS-1 sh-*MECP2*-3, 1.14 ± 0.49; MDS-1 sh-*MECP2*-4, 0.56 ± 0.12. Statistical significance was determined using Tukey’s multiple comparison test (∗*p* < 0.05, ∗∗*p* < 0.01, ∗∗∗*p* < 0.001, ∗∗∗∗*p* < 0.0001). (B and D) Representative images of iPSC-derived neurons stained with anti-MAP2 (green) antibody at 7 DIV after infection with lentiviruses expressing shRNAs. Scale bars: 20 μm (B), 50 μm (D). (C) Representative images of iPSC-derived neurons stained with anti-p-S6 (red) antibody and Hoechst 33258 (blue) at 7 DIV after infection with lentiviruses expressing shRNAs. Scale bars: 100 μm. (E–G) Quantification of neuronal soma size (E), mTOR activity (F), and total dendrite length (G) was performed at 7 DIV using iPSC-derived neurons from two lines: NC-1 (201B7) and MDS-1 (AC1140206AS). Data are presented as mean ± SD from three independent experiments (*n* = 3). In all experiments, at least 50 neurons were analyzed per condition. The average relative soma size was 1.00 ± 0.11 in NC-1 sh-Scramble, 0.58 ± 0.13 in NC-1 sh-*MECP2*-4, 1.67 ± 0.27 in MDS-1 sh-Scramble, and 1.16 ± 0.07 in MDS-1 sh-*MECP2*-4. Relative dendrite length was 1.00 ± 0.10 in NC-1 sh-Scramble, 0.55 ± 0.10 in NC-1 sh-*MECP2*-4, 1.71 ± 0.17 in MDS-1 sh-Scramble, and 1.19 ± 0.14 in MDS-1 sh-*MECP2*-4. p-S6-positive cells accounted for 41.0% ± 3.9% of Hoechst-stained cells in NC-1 sh-Scramble, 21.9% ± 3.4% in NC-1 sh-*MECP2*-4, 62.8% ± 8.7% in MDS-1 sh-Scramble, and 43.7% ± 3.2% in MDS-1 sh-*MECP2*-4. Statistical significance was determined using Tukey’s multiple comparison test (∗*p* < 0.05, ∗∗*p* < 0.01, ∗∗∗*p* < 0.001).

In light of the above results, we investigated the effect of *Mecp2* downregulation on the abnormal phenotypes of MDS neurons. To select the optimal shRNA for *Mecp2* knockdown, we prepared three constructs, sh-*Mecp2*-1, sh-*Mecp2*-2, and sh-*Mecp2*-3, and checked their knockdown efficiency using RT-qPCR analysis. Among them, sh-*Mecp2*-1 and sh-*Mecp2*-2 significantly reduced *Mecp2* expression in both WT and Tg1 mouse neurons (Figure 2A). Primary hippocampal neurons from WT and Tg1 mice were infected with lentiviruses expressing sh-*Mecp2*-1, sh-*Mecp2*-2, or sh-Scramble used as a control. Morphological analysis at 7 DIV revealed that *Mecp2* knockdown in WT neurons reduced neuronal soma size, mTOR signaling activity, and dendritic outgrowth (Figures 2B–2G), suggesting that these phenotypes were affected in opposite directions by increase and decrease in *Mecp2* levels. Similarly, in Tg1 neurons, *Mecp2* knockdown attenuated soma enlargement, mTOR hyperactivation, and excessive dendritic growth (Figures 2B–2G). Human MDS is typically caused by tandem duplication of *MECP2* and its surrounding genes, including the interleukin-1 receptor-associated kinase gene.^32^^,^^33^^,^^34^ In contrast, Tg1 mice only harbor the *Mecp2* gene region, which limits their utility in studying this disease. To clarify the contribution of *MECP2* overexpression to the abnormal phenotypes of MDS neurons, we performed knockdown of *MECP2* in the iPSC neurons derived from patients with MDS. We tested three sh-RNAs targeting human *MECP2*, sh-*MECP2*-2, sh-*MECP2*-3, and sh-*MECP2*-4 and identified sh-*MECP2*-4 as the most effective in reducing *MECP2* expression in neurons derived from both NC and MDS iPSC lines (Figure 3A). iPSC-derived neurons from MDS patients and NC were infected with lentivirus expressing sh-*MECP2*-4 at terminal differentiation. The morphological analysis was performed 7 days post differentiation. *MECP2* knockdown in MDS iPSC-derived neurons significantly attenuated neuronal soma enlargement, mTOR hyperactivation, and dendritic overgrowth (Figures 3B–3G). These results indicate that elevated levels of MeCP2 due to *MECP2* duplication contribute to the abnormal neuronal phenotypes observed in MDS.

### Inhibition of miR-199a improves the phenotypes of MDS model neurons *in vitro*

**Figure 4:**
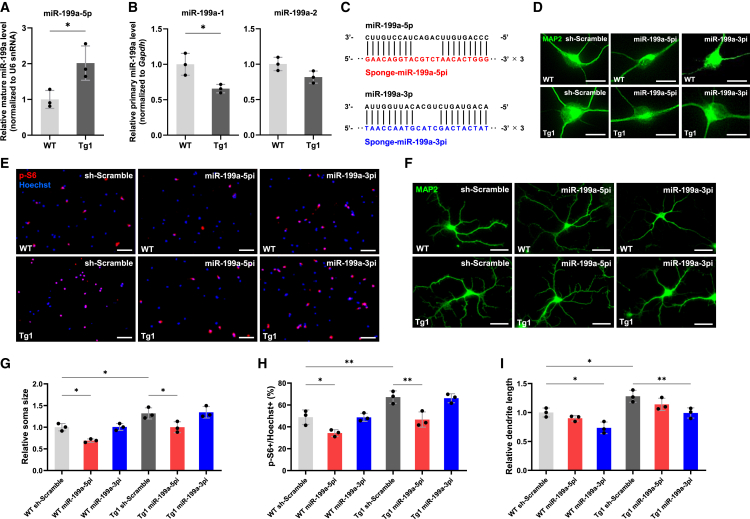
miR-199a contributes to the pathological morphogenesis of hippocampal neurons in Tg1 mice (A) Expression levels of mature miR-199a-5p were measured by RT-qPCR in mouse hippocampal neurons. Neurons were cultured for 7 days and subsequently lysed for RT-qPCR analysis. Data are presented as mean ± SD from three independent experiments (*n* = 3). The relative expression levels of mature miR-199a-5p (normalized to U6 snRNA) were: WT, 1.00 ± 0.25; Tg1, 2.02 ± 0.48. Statistical significance was determined using Student’s t test (∗*p* < 0.05). (B) Expression levels of primary miR-199a-1 and primary miR-199a-2 were measured by RT-qPCR in mouse hippocampal neurons. Neurons were cultured for 7 days and subsequently lysed for RT-qPCR analysis. Data are presented as mean ± SD from three independent experiments (*n* = 3). The relative expression levels of primary miR-199a-1 and primary miR-199a-2 (normalized to *Gapdh*) were: primary miR-199a-1: WT, 1.00 ± 0.15; Tg1, 0.66 ± 0.06; primary miR-199a-2: WT, 1.00 ± 0.09; Tg1, 0.81 ± 0.09. Statistical significance was determined using Student’s t test (∗*p* < 0.05). (C) Diagrams of lentiviral vector constructs expressing sponges against miR-199a-5p and miR-199a-3p. (D and F) Representative images of mouse hippocampal neurons stained with anti-MAP2 (green) antibody at 7 DIV after infection with lentiviruses expressing sponges. Scale bars: 20 μm (D), 50 μm (F). (E) Representative images of mouse hippocampal neurons stained with anti-p-S6 (red) antibody and Hoechst 33258 (blue) at 7 DIV after infection with lentiviruses expressing sponges. Scale bars: 100 μm. (G–I) Quantification of neuronal soma size (G), mTOR activity (H), and total dendrite length (I) at 7 DIV. Data are presented as mean ± SD from three independent experiments (*n* = 3). In all experiments, at least 50 neurons were analyzed per condition. The average relative soma size was 1.00 ± 0.08 in WT sh-Scramble, 0.69 ± 0.04 in WT sponge-miR-199a-5pi, 1.01 ± 0.08 in WT sponge-miR-199a-3pi, 1.32 ± 0.13 in Tg1 sh-Scramble, 0.99 ± 0.13 in Tg1 sponge-miR-199a-5pi, and 1.34 ± 0.13 in Tg1 sponge-miR-199a-3pi. Relative dendrite length was 1.00 ± 0.08 in WT sh-Scramble, 0.90 ± 0.05 in WT sponge-miR-199a-5pi, 0.73 ± 0.10 in WT sponge-miR-199a-3pi, 1.28 ± 0.10 in Tg1 sh-Scramble, 1.14 ± 0.11 in Tg1 sponge-miR-199a-5pi, and 0.99 ± 0.09 in Tg1 sponge-miR-199a-3pi. p-S6-positive cells accounted for 48.8% ± 6.6% of Hoechst-stained cells in WT sh-Scramble, 34.3% ± 3.1% in WT sponge-miR-199a-5pi, 48.7% ± 3.7% in WT sponge-miR-199a-3pi, 67.1% ± 5.7% in Tg1 sh-Scramble, 46.7% ± 6.9% in Tg1 sponge-miR-199a-5pi, and 66.1% ± 4.1% in Tg1 sponge-miR-199a-3pi. Statistical significance was determined using Tukey’s multiple comparison test (∗*p* < 0.05, ∗∗*p* < 0.01).

**Figure 5:**
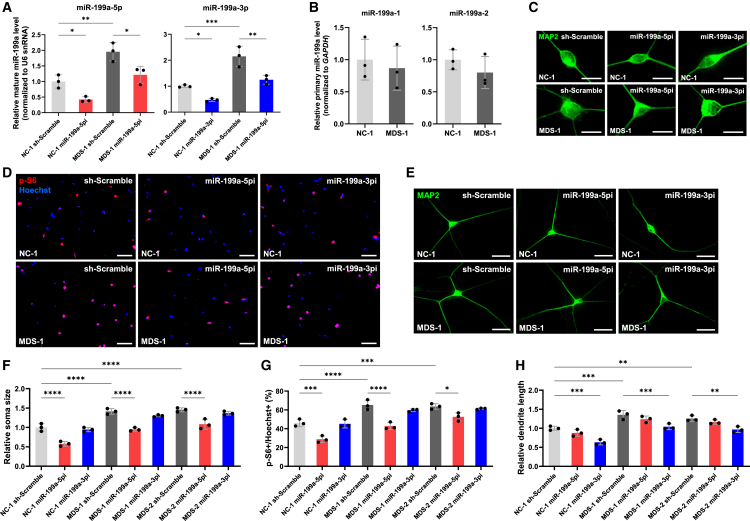
miR-199a contributes to the pathological morphogenesis of iPSC-derived neurons in patients with MDS (A) Expression levels of mature miR-199a-5p and miR-199a-3p were measured by RT-qPCR in human iPSC-derived neurons. Neurons were infected with lentiviruses expressing sponges and cultured for 7 days, then subsequently lysed for RT-qPCR analysis. Data are presented as mean ± SD from three independent experiments (*n* = 3). The relative expression levels of mature miR-199a-5p (normalized to U6 snRNA) were: 1.00 ± 0.21 (sh-Scramble) and 0.42 ± 0.08 (sponge-miR-199a-5pi) in NC-1 neurons, and 1.95 ± 0.30 (sh-Scramble) and 1.21 ± 0.27 (sponge-miR-199a-5pi) in MDS-1 neurons. The relative expression levels of mature miR-199a-3p were: 1.00 ± 0.04 (sh-Scramble) and 0.47 ± 0.06 (sponge-miR-199a-3pi) in NC-1 neurons, and 2.15 ± 0.39 (sh-Scramble) and 1.25 ± 0.17 (sponge-miR-199a-3pi) in MDS-1 neurons. Statistical significance was determined using Tukey’s multiple comparison test (∗*p* < 0.05, ∗∗*p* < 0.01, ∗∗∗*p* < 0.001). (B) Expression levels of primary miR-199a-1 and primary miR-199a-2 were measured by RT-qPCR in human iPSC-derived neurons cultured for 7 days after differentiation. Data are presented as mean ± SD from three independent experiments (*n* = 3). The relative expression levels of primary miR-199a-1 and primary miR-199a-2 (normalized to *GAPDH*) were: primary miR-199a-1: NC-1, 1.00 ± 0.32; MDS-1, 0.87 ± 0.34; primary miR-199a-2: NC-1, 1.00 ± 0.16; MDS-1, 0.80 ± 0.25. Statistical significance was determined using Student’s t test. (C and E) Representative images of human iPSC-derived neurons stained with anti-MAP2 (green) antibody at 7 DIV after infection with lentiviruses expressing sponges. Scale bars: 20 μm (C), 50 μm (E). (D) Representative images of iPSC-derived neurons stained with anti-p-S6 (red) antibody and Hoechst 33258 (blue) at 7 DIV after infection with lentiviruses expressing sponges. Scale bars: 100 μm. (F–H) Quantification of neuronal soma size (F), mTOR activity (G), and total dendrite length (H) was performed at 7 DIV using iPSC-derived neurons from three lines: NC-1, MDS-1, and MDS-2. Data are presented as mean ± SD from three independent experiments (*n* = 3). In all experiments, at least 52 neurons were analyzed per condition. The average relative soma size was 1.00 ± 0.10 in NC-1 sh-Scramble, 0.58 ± 0.06 in NC-1 sponge-miR-199a-5pi, 0.94 ± 0.05 in NC-1 sponge-miR-199a-3pi, 1.41 ± 0.07 in MDS-1 sh-Scramble, 0.95 ± 0.04 in MDS-1 sponge-miR-199a-5pi, 1.30 ± 0.03 in MDS-1 sponge-miR-199a-3pi, 1.46 ± 0.05 in MDS-2 sh-Scramble, 1.08 ± 0.13 in MDS-2 sponge-miR-199a-5pi, and 1.37 ± 0.05 in MDS-2 sponge-miR-199a-3pi. Relative dendrite length was 1.00 ± 0.05 in NC-1 sh-Scramble, 0.88 ± 0.09 in NC-1 sponge-miR-199a-5pi, 0.63 ± 0.07 in NC-1 sponge-miR-199a-3pi, 1.35 ± 0.11 in MDS-1 sh-Scramble, 1.24 ± 0.08 in MDS-1 sponge-miR-199a-5pi, 1.04 ± 0.08 in MDS-1 sponge-miR-199a-3pi, 1.26 ± 0.07 in MDS-2 sh-Scramble, 1.16 ± 0.06 in MDS-1 sponge-miR-199a-5pi, and 0.97 ± 0.08 in MDS-2 sponge-miR-199a-3pi. p-S6-positive cells accounted for 46.1% ± 3.8% of Hoechst-stained cells in NC-1 sh-Scramble, 28.8% ± 3.6% in NC-1 sponge-miR-199a-5pi, 45.2% ± 4.4% in NC-1 sponge-miR-199a-3pi, 65.2% ± 5.3% in MDS-1 sh-Scramble, 42.8% ± 3.9% in MDS-1 sponge-miR-199a-5pi, 59.6% ± 1.4% in MDS-1 sponge-miR-199a-3pi, 63.6% ± 2.9% in MDS-2 sh-Scramble, 52.6% ± 4.4% in MDS-2 sponge-miR-199a-5pi, and 61.3% ± 0.78% in MDS-2 sponge-miR-199a-3pi. Statistical significance was determined using Tukey’s multiple comparison test (∗*p* < 0.05, ∗∗*p* < 0.01).

**Figure 6:**
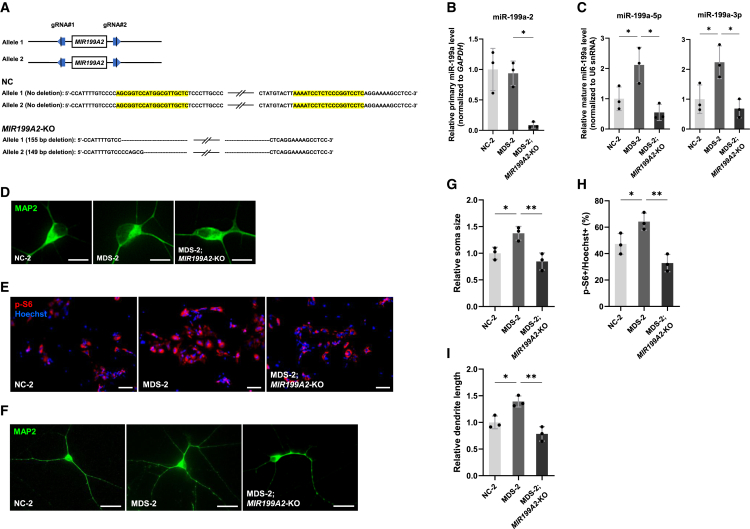
CRISPR-Cas9-mediated knockout of *MIR199A2* attenuates neuronal abnormalities in MDS neurons (A) Schematic of CRISPR-Cas9-mediated homozygous deletion of *MIR199A2* in MDS-2 iPSCs (MDS-2; *MIR199A2*-KO). The gRNA target sequences are highlighted in yellow. Allele 1 carries a 155-bp deletion, and Allele 2 carries a 149-bp deletion. (B and C) RT-qPCR validation of homozygous knockout in isogenic MDS-2; *MIR199A2*-KO neurons at 7 DIV, showing reduced expression of primary miR-199a-2 (B, normalized to *GAPDH*) and mature miR-199a-5p and miR-199a-3p (C, normalized to U6 snRNA). Data are presented as mean ± SD from three independent experiments (*n* = 3). The relative expression levels of primary miR-199a-2 were 1.00 ± 0.34 (NC-2), 0.93 ± 0.20 (MDS-2), and 0.08 ± 0.05 (MDS-2; *MIR199A2*-KO). The relative expression levels of mature miR-199a-5p were 1.00 ± 0.39 (NC-2), 2.11 ± 0.58 (MDS-2), and 0.54 ± 0.26 (MDS-2; *MIR199A2*-KO), and those of miR-199a-3p were 1.00 ± 0.47 (NC-2), 2.23 ± 0.54 (MDS-2), and 0.68 ± 0.31 (MDS-2; *MIR199A2*-KO). Statistical significance was determined using Tukey’s multiple comparison test (∗*p* < 0.05). (D and F) Representative images of neurons differentiated from NC-2, MDS-2, and MDS-2; *MIR199A2*-KO iPSCs stained with anti-MAP2 (green) antibody at 7 DIV. Scale bars: 20 μm (D), 50 μm (F). (E) Representative images of neurons differentiated from NC-2, MDS-2, and MDS-2; *MIR199A2*-KO iPSCs stained with anti-p-S6 (red) antibody and Hoechst 33258 (blue) at 7 DIV. Scale bars: 100 μm. (G–I) Quantification of neuronal soma size (G), mTOR activity (H), and total dendrite length (I) was performed at 7 DIV using neurons differentiated from NC-2, MDS-2, and MDS-2; *MIR199A2*-KO iPSCs. Data are presented as mean ± SD from three independent experiments (*n* = 3). In all experiments, at least 55 neurons were analyzed per condition. The average relative soma size was 1.00 ± 0.11 in NC-2, 1.37 ± 0.12 in MDS-2, and 0.84 ± 0.16 in MDS-2; *MIR199A2*-KO neurons. Relative dendrite length was 1.00 ± 0.11 in NC-2, 1.39 ± 0.10 in MDS-2, and 0.78 ± 0.13 in MDS-2; *MIR199A2*-KO neurons. The proportion of p-S6-positive cells among Hoechst-stained cells was 47.3% ± 7.9% in NC-2, 64.2% ± 6.4% in MDS-2, and 32.8% ± 6.5% in MDS-2; *MIR199A2*-KO neurons. Statistical significance was determined using Tukey’s multiple comparison test (∗*p* < 0.05, ∗∗*p* < 0.01).

We previously demonstrated that MeCP2 promotes post-transcriptional processing of miR-199a as a component of the Drosha complex.^8^ Overexpression of MeCP2 in WT mouse neurons significantly increased mature miR-199a levels, while the expression of primary transcripts remained unchanged.^8^ To further explore the role of the MeCP2/miR-199a axis in the pathophysiology of MDS, we first quantified mature miR-199a levels in Tg1 mouse neurons. The levels of mature miR-199a were significantly elevated in Tg1 neurons (Figure 4A), whereas primary miR-199a-1 and -2 transcripts were either slightly reduced or unchanged compared to WT neurons (Figure 4B), suggesting that post-transcriptional regulation accounts for the increased mature miR-199a levels. Consistent with our previous findings, MeCP2 likely enhances Drosha-mediated processing of primary-miR-199a, thereby promoting the biogenesis of mature miR-199a without altering primary miR-199a levels.^8^ To investigate the functional impact of miR-199a, we employed miRNA sponges that mimic the effects of competing with endogenous RNA and depleting the available miRNA pool.^35^ We previously designed miRNA sponges targeting mature forms of miR-199a: miR-199a-5p and miR-199a-3p (Figure 4C).^8^ Primary hippocampal neurons of Tg1 and WT mice were infected with lentiviruses expressing miRNA sponges against either miR-199a-5p (sponge-miR-199a-5pi) or miR-199a-3p (sponge-miR-199a-3pi), and morphological analysis was performed at 7 DIV. Inhibition of miR-199a-5p significantly reduced MeCP2-mediated neuronal soma growth and mTOR signaling activity, while inhibition of miR-199a-3p had minimal effect on neuronal soma size and mTOR activity in mouse neurons (Figures 4D, 4E, 4G, and 4H). In contrast, miR-199a-3p inhibition markedly reduced dendritic overgrowth, with little effect on miR-199a-5p inhibition (Figures 4F and 4I). We next assessed the expression of miR-199a in iPSC-derived neurons from MDS patients. Similar to the mouse model, mature miR-199a levels were significantly increased in patient-derived neurons, while primary transcript levels remained unchanged (Figures 5A and 5B). Lentiviral delivery of miR-199a-5p or -3p sponges effectively reduced the levels of the corresponding mature miRNAs (Figure 5A) and attenuated abnormal soma enlargement, mTOR hyperactivation, and dendritic overgrowth (Figures 5C–5H). To further validate the contribution of miR-199a to the abnormal neuronal phenotypes observed in MDS, we generated an isogenic iPSC line in which the *MIR199A2* locus was homozygously knocked out on the MDS iPSC background (MDS; *MIR199A2*-KO) using CRISPR-Cas9 genome editing (Figure 6A). We differentiated both the original isogenic MDS iPSCs and the MDS; *MIR199A2*-KO iPSCs into neurons and analyzed neuronal morphology and mTOR signaling activity. The KO was validated by qPCR, which showed a marked reduction in the expression of primary miR-199a-2 as well as mature miR-199a-5p and -3p (Figures 6B and 6C). Compared with original MDS neurons, neurons differentiated from MDS; *MIR199A2*-KO iPSCs exhibited significantly smaller soma size and reduced dendritic length, along with attenuated mTOR signaling activity, as indicated by the decreased proportion of p-S6-positive cells (Figures 6D–6I). These results are consistent with the findings from the miRNA sponge-mediated knockdown experiments and provide genetic evidence that miR-199a plays a critical role in mediating the aberrant neuronal morphology and mTOR hyperactivation in MDS. Taken together, our findings support the involvement of miR-199a in the pathophysiology of MDS as a downstream effector of MeCP2.

### Decreased expression of miR-199a improves abnormal mTOR signaling activity and synaptic density in MDS model mice

**Figure 7:**
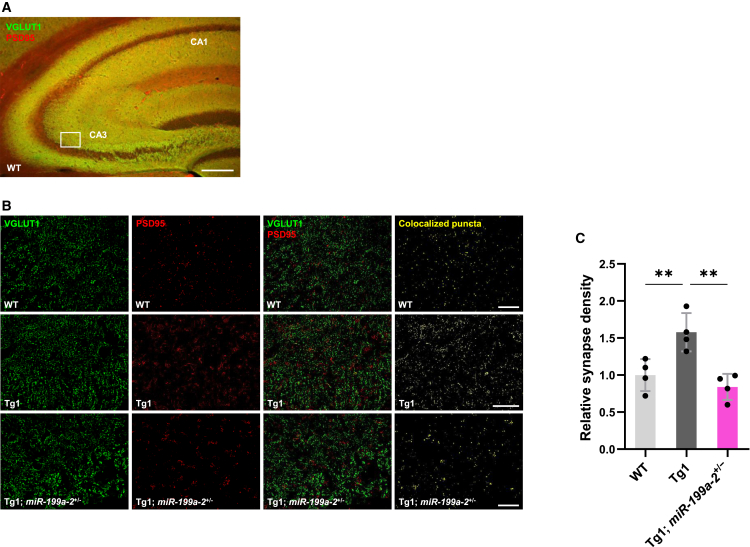
Reduction of miR-199a alleviates excitatory synaptic hyperdensity in Tg1 mice (A) Hippocampus was immunostained for VGLUT1 (green) and PSD95 (red) at the age of 6 weeks. Scale bar: 200 μm. (B) Representative immunohistological images of hippocampus CA3 regions. Brain sections at the age of 6 weeks were stained with anti-VGLUT1 (green) and PSD95 (red) antibodies. Colocalized signals (yellow) of VGLUT1 and PSD95 were detected. Scale bars: 20 μm. (C) Quantification of glutamatergic synapse density. Data are presented as mean ± SD from four mice (six regions per mouse, *n* = 4). The average relative synapse density was: WT, 1.00 ± 0.22; Tg1, 1.58 ± 0.26; Tg1; miR-199a-2^+^/^−^, 0.84 ± 0.18. Statistical significance was determined using Tukey’s multiple comparison test (∗*p* < 0.05, ∗∗*p* < 0.01).

**Figure 8:**
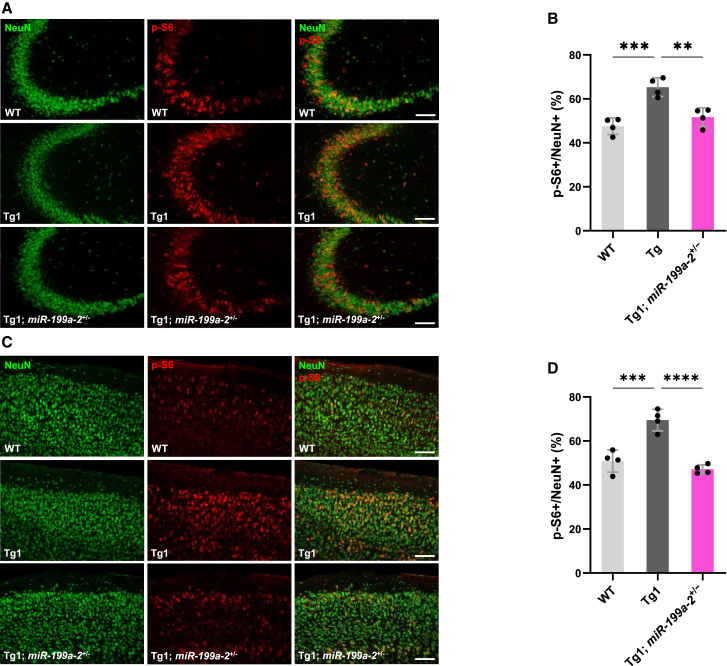
Reduction of miR-199a ameliorates the abnormal hyperactivity of mTOR in the brain of Tg1 mice (A) Representative immunohistological images of hippocampus CA3 regions. Brain sections at 6 weeks were stained with anti-NeuN (green) and p-S6 (red) antibodies. Scale bars: 100 μm. (B) Quantification of mTOR signaling activity in the hippocampus CA3 region, shown as the percentage of p-S6-positive cells among NeuN-positive neurons. Data are presented as mean ± SD from four mice (*n* = 4). In all experiments, at least 146 NeuN-positive neurons per mouse were analyzed. The average percentage of p-S6-positive cells was: WT, 45.1% ± 4.2%; Tg1, 67.8% ± 5.1%; Tg1; miR-199a-2^+^/^−^, 52.6% ± 4.5%. Statistical significance was determined using Tukey’s multiple comparison test (∗*p* < 0.05, ∗∗*p* < 0.01). (C) Representative immunohistological images of cortices. Brain sections at 6 weeks were stained with anti-NeuN (green) and p-S6 (red) antibodies. Scale bars: 100 μm. (D) Quantification of mTOR signaling activity in the cortex, shown as the percentage of p-S6- positive cells among NeuN-positive neurons. Data are presented as mean ± SD from four independent mice (*n* = 4). In all experiments, at least 186 NeuN-positive neurons per mouse were analyzed. The average percentage of p-S6-positive cells was: WT, 50.8% ± 5.0%; Tg1, 69.5% ± 4.9%; Tg1; miR-199a-2^+^/^−^, 47.2% ± 2.0%. Statistical significance was determined using Tukey’s multiple comparison test (∗∗*p* < 0.01, ∗∗∗*p* < 0.001).

miR-199a is encoded in two distinct genomic loci: *miR-199a-1* on chromosome 19 and *miR-199a-2* on chromosome 1. Previous reports have indicated that *miR-199a-1* KO mice exhibit normal viability and phenotype (Jackson Laboratory strain #017512), whereas *miR-199a-2* KO mice can recapitulate many RTT-like features.^8^ These include growth retardation, motor abnormalities, hypoactivity, and early lethality. Neurons also exhibit reduced soma size and increased packing density, resembling phenotypes observed in RTT patients and *Mecp2*-KO mice. Our previous findings showed that deletion of *miR-199a-2* markedly reduces the expression of both miR-199a-5p and miR-199a-3p, with levels lower than those observed in *Mecp2*-KO mice, and with minimal compensatory changes in *miR-199a-1*.^8^ Therefore, we selected *miR-199a-2* KO mice to investigate the role of miR-199a downregulation *in vivo* within the MDS mouse model context. To explore the contribution of miR-199a to MDS pathophysiology, we crossed Tg1 mice with *miR-199a-2* heterozygous KO mice (*miR-199a-2*^+/−^). Alterations in MeCP2 expression are known to affect synaptic density in the mouse hippocampus, with *MECP2* duplication increasing glutamatergic synapse numbers.^36^ To assess glutamatergic synaptic density, we performed immunostaining and quantitative colocalization analysis of VGLUT1 (a presynaptic marker) and PSD95 (a postsynaptic marker) in the hippocampal CA3 region of 6-week-old mice (Figure 7A). Tg1 mice displayed a significant increase in the density of colocalized VGLUT1-PSD95 puncta compared to WT mice. Importantly, heterozygous deletion of *miR-199a-2* significantly attenuated the enhanced glutamatergic synaptic density observed in Tg1 mice (Figures 7B and 7C). Furthermore, we investigated whether reduction of miR-199a could alleviate abnormal mTOR activation observed in Tg1 mice *in vivo*. Consistent with our *in vitro* results showing that miR-199a inhibition attenuates mTOR hyperactivation, immunohistochemical analyses revealed significantly decreased numbers of p-S6-positive neurons in both the cerebral cortex and hippocampus of Tg1; *miR-199a-2*^+/−^ mice compared with Tg1 mice (Figures 8A–8D). Taken together, these findings suggest that the MeCP2/miR-199a pathway plays a significant role in the neuronal abnormalities associated with MDS.

### PDE4D and QKI function as distinct downstream targets of miR-199a-5p and miR-199a-3p in MDS model neurons

**Figure 9:**
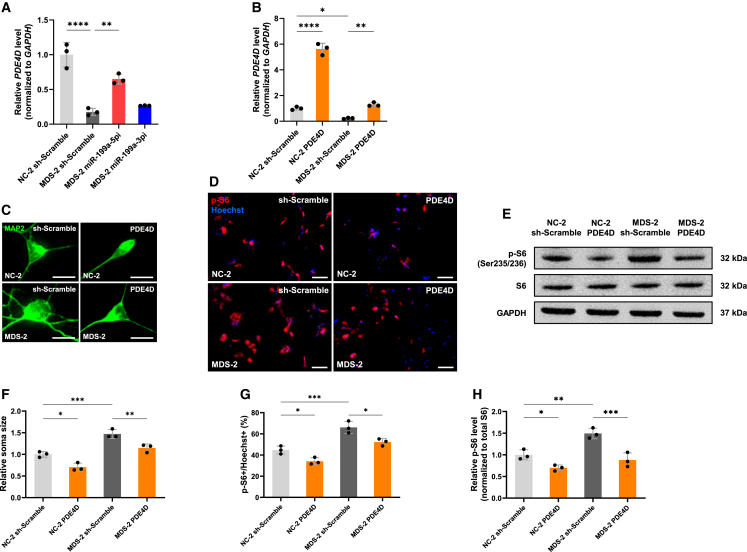
miR-199a-5p-mediated suppression of *PDE4D* contributes to mTOR hyperactivation in MDS neurons (A) Expression levels of *PDE4D* were measured by RT-qPCR in human iPSC-derived neurons. Neurons were infected with lentiviruses expressing miR-199a-5p or miR-199a-3p sponges and cultured for 7 days, followed by RNA extraction for RT-qPCR analysis. Data are presented as mean ± SD from three independent experiments (*n* = 3). The relative expression levels of *PDE4D* (normalized to *GAPDH*) were: NC-2 sh-Scramble, 1.00 ± 0.18; MDS-2 sh-Scramble, 0.18 ± 0.05; MDS-2 sponge-miR-199a-5pi, 0.65 ± 0.07; MDS-2 sponge-miR-199a-3pi, 0.27 ± 0.01. Statistical significance was determined using Tukey’s multiple comparison test (∗∗*p* < 0.01, ∗∗∗∗*p* < 0.0001). (B) Expression levels of *PDE4D* were measured by RT-qPCR in human iPSC-derived neurons to confirm the efficiency of *PDE4D* overexpression. Neurons were infected with lentiviruses expressing *PDE4D* and cultured for 7 days before RNA extraction. Data are presented as mean ± SD from three independent experiments (*n* = 3). The relative expression levels of *PDE4D* (normalized to *GAPDH*) were: NC-2 sh-Scramble, 1.00 ± 0.12; NC-2 PDE4D, 5.62 ± 0.44; MDS-2 sh-Scramble, 0.23 ± 0.04; MDS-2 PDE4D, 1.30 ± 0.15. Statistical significance was determined using Tukey’s multiple comparison test (∗*p* < 0.05, ∗∗*p* < 0.01, ∗∗∗∗*p* < 0.0001). (C) Representative images of human iPSC-derived neurons stained with anti-MAP2 (green) antibody at 7 DIV after infection with lentiviruses expressing *PDE4D*. Scale bars: 20 μm. (D) Representative images of iPSC-derived neurons stained with anti-p-S6 (red) antibody and Hoechst 33258 (blue) at 7 DIV after infection with lentiviruses expressing *PDE4D*. Scale bars: 100 μm. (E) Representative Western blot images of p-S6, total S6, and GAPDH in human iPSC-derived neurons at 7 DIV. Neurons were infected with lentiviruses expressing *PDE4D* and lysed for immunoblotting. The p-S6/S6 ratio was used to evaluate mTOR activity, and GAPDH was used as a loading control. (F and G) Quantification of neuronal soma size (F) and mTOR activity (G) at 7 DIV using iPSC-derived neurons from two lines: NC-2 and MDS-2. Neurons were infected with lentiviruses expressing *PDE4D* and analyzed by immunocytochemistry. Data are presented as mean ± SD from three independent experiments (*n* = 3). At least 53 neurons were analyzed per condition. The average relative soma size was 1.00 ± 0.07 in NC-2 sh-Scramble, 0.70 ± 0.09 in NC-2 PDE4D, 1.47 ± 0.10 in MDS-2 sh-Scramble, and 1.15 ± 0.09 in MDS-2 PDE4D. p-S6-positive cells accounted for 44.8% ± 3.8% of Hoechst-stained cells in NC-2 sh-Scramble, 34.1% ± 3.3% in NC-2 PDE4D, 66.1% ± 5.7% in MDS-2 sh-Scramble, and 52.2% ± 3.4% in MDS-2 PDE4D. Statistical significance was determined using Tukey’s multiple comparison test (∗*p* < 0.05, ∗∗∗*p* < 0.001). (H) Quantification of mTOR activity based on p-S6/S6 ratio from Western blot analysis at 7 DIV using iPSC-derived neurons from two lines: NC-2 and MDS-2. Neurons were infected with lentiviruses expressing *PDE4D*. Data are presented as mean ± SD from three independent experiments (*n* = 3). The average relative p-S6/S6 ratio was 1.00 ± 0.12 in NC-2 sh-Scramble, 0.70 ± 0.07 in NC-2 PDE4D, 1.49 ± 0.12 in MDS-2 sh-Scramble, and 0.88 ± 0.17 in MDS-2 PDE4D. Statistical significance was determined using Tukey’s multiple comparison test (∗*p* < 0.05, ∗∗*p* < 0.01, ∗∗∗*p* < 0.001).

Inhibition of miR-199a-5p significantly reduced neuronal soma size and mTOR signaling activity, whereas miR-199a-3p inhibition had a limited effect on these parameters. In contrast, dendritic outgrowth was markedly suppressed by miR-199a-3p inhibition. These findings suggest that miR-199a-5p and miR-199a-3p exert distinct effects on MDS neuronal phenotypes, likely via regulation of different target genes. We previously demonstrated that cAMP-specific 3′,5′-cyclic phosphodiesterase 4D (*PDE4D*) is a direct target of miR-199a-5p in MeCP2-deficient neurons.^8^ *PDE4D* functions as a negative regulator of mTOR signaling.^37^ To investigate whether *PDE4D* contributes to the pathophysiology of MDS, we examined its expression and functional relevance in iPSC-derived neurons. RT-qPCR analysis revealed that *PDE4D* expression was reduced in MDS neurons and partially restored by miR-199a-5p inhibition (Figure 9A). To assess whether restoring *PDE4D* expression could ameliorate MDS-associated abnormalities, *PDE4D* was overexpressed in neurons using lentiviral transduction, and overexpression efficiency was confirmed by RT-qPCR (Figure 9B). *PDE4D* overexpression, which decreased soma size and mTOR activity in NC neurons, reduced soma size and the number of p-S6-positive cells in MDS neurons, as evaluated by immunocytochemistry (Figures 9C, 9D, 9F, and 9G). Western blot analysis further demonstrated that *PDE4D* overexpression decreased p-S6/S6 ratios in NC and MDS neurons, indicating suppression of mTOR activity (Figures 9E and 9H). These findings support the role of *PDE4D* downregulation in promoting mTOR hyperactivation and morphological abnormalities in MDS neurons.

**Figure 10:**
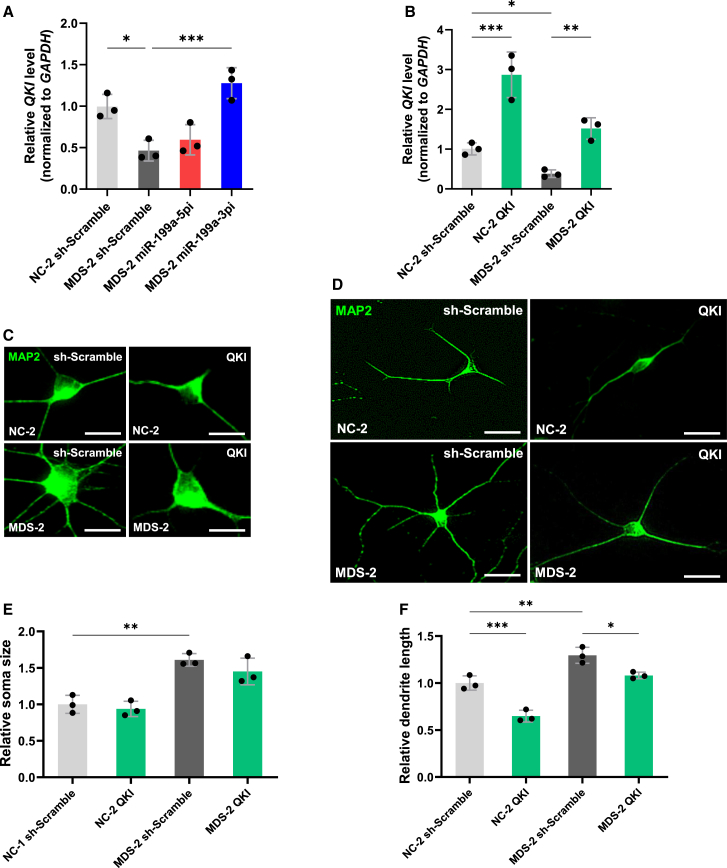
miR-199a-3p-mediated suppression of *QKI* leads to dendritic overgrowth in MDS neurons (A) Expression levels of *QKI* were measured by RT-qPCR in human iPSC-derived neurons. Neurons were infected with lentiviruses expressing miR-199a-5p or miR-199a-3p sponges and cultured for 7 days, followed by RNA extraction for RT-qPCR analysis. Data are presented as mean ± SD from three independent experiments (*n* = 3). The relative expression levels of *QKI* (normalized to *GAPDH*) were: NC-2 sh-Scramble, 1.00 ± 0.14; MDS-2 sh-Scramble, 0.46 ± 0.12; MDS-2 sponge-miR-199a-5pi, 0.60 ± 0.18; MDS-2 sponge-miR-199a-3pi, 1.28 ± 0.19. Statistical significance was determined using Tukey’s multiple comparison test (∗*p* < 0.05, ∗∗∗*p* < 0.001). (B) Expression levels of *QKI* were measured by RT-qPCR in human iPSC-derived neurons to confirm the efficiency of *QKI* overexpression. Neurons were infected with lentiviruses expressing *QKI* and cultured for 7 days before RNA extraction. Data are presented as mean ± SD from three independent experiments (*n* = 3). The relative expression levels of *QKI* (normalized to *GAPDH*) were: NC-2 sh-Scramble, 1.00 ± 0.15; NC-2 QKI, 2.87 ± 0.57; MDS-2 sh-Scramble, 0.38 ± 0.09; MDS-2 QKI, 1.52 ± 0.07. Statistical significance was determined using Tukey’s multiple comparison test (∗*p* < 0.05, ∗∗*p* < 0.01, ∗∗∗*p* < 0.001). (C and D) Representative images of human iPSC-derived neurons stained with anti-MAP2 (green) antibody at 7 DIV after infection with lentiviruses expressing *QKI*. Scale bars: 20 μm (C), 50 μm (D). (E and F) Quantification of neuronal soma size (E) and total dendrite length (F) was performed at 7 DIV using iPSC-derived neurons from two lines: NC-2 and MDS-2. Data are presented as mean ± SD from three independent experiments (*n* = 3). In all experiments, at least 53 neurons were analyzed per condition. The average relative soma size was 1.00 ± 0.12 in NC-2 sh-Scramble, 0.94 ± 0.10 in NC-2 QKI, 1.61 ± 0.09 in MDS-2 sh-Scramble, and 1.45 ± 0.18 in MDS-2 QKI. Relative dendrite length was 1.00 ± 0.08 in NC-2 sh-Scramble, 0.65 ± 0.06 in NC-2 QKI, 1.30 ± 0.09 in MDS-2 sh-Scramble, and 1.08 ± 0.04 in MDS-2 QKI. Statistical significance was determined using Tukey’s multiple comparison test (∗*p* < 0.05, ∗∗*p* < 0.01, ∗∗∗*p* < 0.001).

Although several targets of miR-199a have been proposed, the downstream effectors of miR-199a-3p in neurons remain incompletely understood. We previously showed that *Quaking* (*Qki*), a gene encoding an RNA-binding protein that regulates neuronal development, suppresses dendritic growth when overexpressed and promotes dendritic growth when knocked down in WT mouse neurons.^38^ More recently, we demonstrated in an RTT mouse model that *Qki* acts downstream of miR-199a-3p and contributes to impaired dendritic growth.^39^ Based on these findings, we investigated whether *QKI* downregulation by miR-199a-3p contributes to the enhanced dendritic growth observed in MDS neurons. We first assessed the *QKI* expression in iPSC-derived neurons. RT-qPCR analysis revealed that *QKI* expression was significantly reduced in MDS neurons and restored by inhibition of miR-199a-3p (Figure 10A). To evaluate the functional relevance of *QKI* downregulation, we overexpressed *QKI* in iPSC-derived neurons using lentiviral vectors, and overexpression efficiency was confirmed by RT-qPCR (Figure 10B). Immunocytochemical analysis showed that *QKI* overexpression significantly attenuated excessive dendritic outgrowth in MDS neurons, and also reduced dendritic length in NC neurons, whereas soma size remained unaffected (Figures 10C–10F). These results indicate that *QKI* is a downstream target of miR-199a-3p and contributes to dendritic abnormalities in MDS neurons.

### Decreased expression of miR-199a improves abnormal neuronal activity in MDS patient-derived cortical organoids

**Figure 11:**
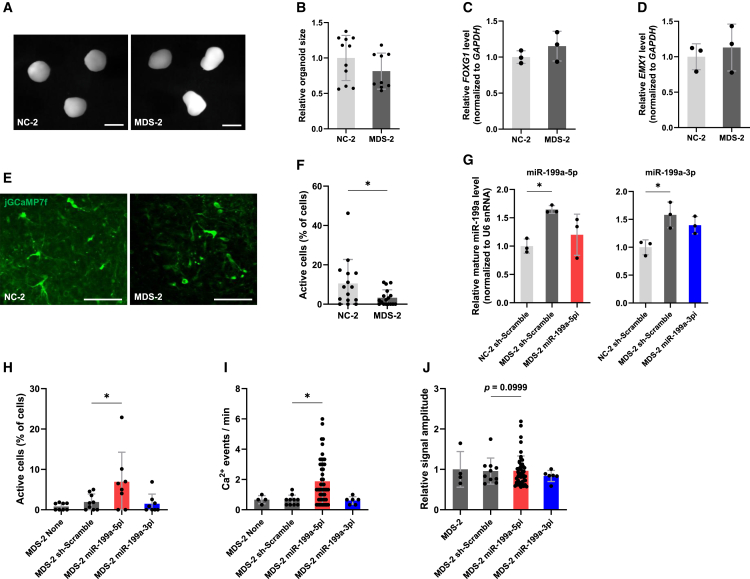
miR-199a-5p inhibition improves neuronal activity in MDS cortical organoids (A) Representative images of cortical organoids at day 50 derived from NC and MDS iPSCs. Scale bars: 2 mm. (B) Quantification of organoid size at day 50. Data are presented as mean ± SD. A total of 11 NC-2 organoids and 9 MDS-2 organoids were analyzed. The average relative organoid size was 1.0 ± 0.32 in NC-2 and 0.81 ± 0.25 in MDS-2. Statistical significance was determined using Student’s t test. (C) Expression levels of *FOXG1* were measured by RT-qPCR in cortical organoids at day 80. Data are presented as mean ± SD from three independent experiments (*n* = 3). The relative expression levels of *FOXG1* (normalized to *GAPDH*) were 1.0 ± 0.088 in NC-2 and 1.2 ± 0.20 in MDS-2. Statistical significance was determined using Student’s t test. (D) Expression levels of *EMX1* were measured by RT-qPCR in cortical organoids at day 80. Data are presented as mean ± SD from three independent experiments (*n* = 3). The relative expression levels of *EMX1* (normalized to *GAPDH*) were 1.0 ± 0.18 in NC-2 and 1.1 ± 0.33 in MDS-2. Statistical significance was determined using Student’s t test. (E) Representative two-photon fluorescence images of neuronal activity in NC and MDS cortical organoids at day 50 expressing jGCaMP7f. Scale bars: 100 μm. (F) Quantification of the active cell rate in NC-2 and MDS-2 cortical organoids at day 50–52. Data are presented as mean ± SD. A total of 15 NC-2 organoids and 17 MDS-2 organoids were analyzed. The average active cell rate was 10.5% ± 12.2% in NC-2 and 3.28% ± 4.06% in MDS-2. Statistical significance was determined using Student’s t test (∗*p* < 0.05). (G) Expression levels of mature miR-199a-5p and miR-199a-3p were measured by RT-qPCR in cortical organoids at day 80. Data are presented as mean ± SD from three independent experiments (*n* = 3). The relative expression levels of mature miR-199a-5p (normalized to U6 snRNA) were: 1.0 ± 0.12 (NC-2 + sh-Scramble), 1.7 ± 0.068 (MDS-2 + sh-Scramble), and 1.2 ± 0.36 (MDS-2 + sponge-miR-199a-5pi). The relative expression levels of mature miR-199a-3p were: 1.0 ± 0.13 (NC-2 + sh-Scramble), 1.6 ± 0.23 (MDS-2 + sh-Scramble), and 1.4 ± 0.15 (MDS-2 + sponge-miR-199a-3pi). Statistical significance was determined using Dunnett’s multiple comparison test (∗*p* < 0.05). (H) Quantification of the active cell rate in cortical organoids at day 80 derived from MDS-2 organoids without infection (None), with control lentivirus (sh-Scramble), or with lentiviruses expressing sponges targeting miR-199a-5p (miR-199a-5pi) or miR-199a-3p (miR-199a-3pi). Data are presented as mean ± SD. A total of 8 organoids for None, 9 organoids for sh-Scramble, 8 organoids for miR-199a-5pi, and 8 organoids for miR-199a-3pi were analyzed. The average active cell rate was 0.820 ± 0.882% in None, 1.93% ± 1.99% in sh-Scramble, 6.95% ± 7.30% in miR-199a-5pi, and 1.50% ± 2.38% in miR-199a-3pi. Statistical significance was determined using Dunnett’s multiple comparison test (∗*p* < 0.05). (I) Quantification of Ca^2+^ transient frequency (events/min) in cortical organoids at day 80 derived from MDS-2 organoids without infection (None), with control lentivirus (sh-Scramble), or with lentiviruses expressing sponges targeting miR-199a-5p (miR-199a-5pi) or miR-199a-3p (miR-199a-3pi). Data are presented as mean ± SD. A total of 4 cells for None, 10 cells for sh-Scramble, 50 cells for sponge-5pi, and 6 cells for sponge-3pi were analyzed. The average Ca^2+^ transient frequency was 0.67 ± 0.27 events/min in None, 0.63 ± 0.33 in sh-Scramble, 1.9 ± 1.5 in miR-199a-5pi, and 0.61 ± 0.25 in miR-199a-3pi. Statistical significance was determined using Dunnett’s multiple comparison test (∗*p* < 0.05). (J) Quantification of Ca^2+^ signal amplitude in cortical organoids at day 80 derived from MDS-2 organoids without infection (None), with control lentivirus (sh-Scramble), or with lentiviruses expressing sponges targeting miR-199a-5p (miR-199a-5pi) or miR-199a-3p (miR-199a-3pi). Data are presented as mean ± SD. A total of 4 cells for None, 10 cells for sh-Scramble, 50 cells for sponge-5pi, and 6 cells for sponge-3pi were analyzed. The relative signal amplitude was 1.0 ± 0.44 in None, 0.96 ± 0.32 in sh-Scramble, 0.97 ± 0.37 in miR-199a-5pi, and 0.84 ± 0.14 in miR-199a-3pi. Statistical significance was determined using Dunnett’s multiple comparison test.

The molecular mechanisms underlying the clinical phenotypes of MDS patients remain largely unknown. Conventional two-dimensional neuronal models cannot fully recapitulate the complex architecture and network activity of the human brain; therefore, we additionally employed three-dimensional cortical organoids to better capture human-specific aspects of MDS pathophysiology. To gain mechanistic insight into the pathogenesis of MDS, we generated cortical organoids from patient-derived iPSCs using a SFEBq-based protocol^40^ and examined their developmental and neuronal characteristics. At 50 DIV, there was no significant difference in organoid size between MDS and NC cortical organoids (Figures 11A and 11B). Furthermore, qPCR analysis at 80 DIV revealed no significant differences in the expression levels of the forebrain markers *FOXG1* and *EMX1* between MDS and NC organoids, indicating comparable forebrain patterning (Figures 11C and 11D). Notably, two-photon calcium imaging performed at 50 DIV revealed a reduction in spontaneous neuronal activity in MDS organoids compared to NC organoids (Figures 11E and 11F). We next examined whether inhibition of miR-199a could rescue the aberrant neuronal activity observed in MDS organoids. To this end, we transduced MDS organoids with lentiviruses expressing sponges targeting miR-199a-5p or miR-199a-3p at 20 DIV and evaluated neuronal activity at 80 DIV. The efficacy of miR-199a inhibition was confirmed by qPCR analysis prior to functional assessment (Figure 11G). Importantly, inhibition of miR-199a-5p partially restored the impaired neuronal activity in MDS organoids, whereas inhibition of miR-199a-3p or expression of a scrambled control had no apparent effect (Figures 11H–11J). These findings support the notion that miR-199a contributes to MeCP2-mediated effects on human cortical development.

## Discussion

Our earlier study showed that miR-199a is a downstream target of MeCP2 in RTT pathogenesis by linking MeCP2 to mTOR signaling. Thus, we hypothesized that *MECP2* overexpression would lead to changes in neuronal structure and signaling opposite to those observed in *MECP2* haploinsufficiency. In this study, we demonstrated that miR-199a is a key mediator of pathological phenotypes in MDS. Using MDS Tg1 mice, patient iPSC-derived neurons, and cortical organoids, we showed that *MECP2* overexpression induced upregulation of miR-199a, resulting in abnormal neuronal morphology, enhanced mTOR activity, altered synaptic density, and impaired neuronal activity. Importantly, inhibition of miR-199a ameliorated these abnormalities, supporting our hypothesis and highlighting the critical role of the MeCP2/miR-199a axis in MDS pathophysiology.

Proper dendritic morphogenesis and synapse formation provide a structural basis for neuronal development and function. Alterations in dendrite morphology and inappropriate synapse formation are implicated in the pathophysiology of various neurological and neurodevelopmental disorders, including ASD and RTT.^41^^,^^42^ Additionally, increased formation of dendritic spines has been observed in several mouse models of autism, including MDS.^43^^,^^44^ Furthermore, neurons overexpressing MeCP2 exhibited an increase in glutamatergic synapse density, which may be involved in the pathophysiology of the autistic features of MDS.^36^ A previous study demonstrated that normalizing MeCP2 levels restored glutamatergic synapse density in *Tg1; Mecp2 null* neurons.^36^ In this study, we found that restoring miR-199a expression rescued aberrant dendritic outgrowth in MDS model neurons *in vitro* and reduced glutamatergic synapse density in *Tg1; miR-199a-2*^*+/−*^ mice *in vivo*. Thus, miR-199a downregulation reversed the pathological phenotypes caused by MeCP2 overexpression, indicating that miR-199a plays a critical functional role downstream of MeCP2 in the pathophysiology of MDS. An earlier study reported that dendritic length was unchanged in cultured hippocampal neurons of Tg1 mice but increased in the Golgi-stained brain sections.^45^ These findings appear to differ from our observations of increased dendritic outgrowth in cultured MDS neurons, which may be attributed to differences in experimental context, neuronal subtypes, or maturation stages. Another recent study using human iPSC-derived neurons reported increased neurite length in MDS models, which was consistent with our observations.^46^

The mTOR signaling pathway is involved in numerous physiological functions in the CNS. It is activated by neurotrophic factors and neurotransmitters to promote protein synthesis and suppress autophagy.^47^ Dysregulation of mTOR is implicated in various neurodevelopmental disorders.^48^ Pathogenic variants of *PTEN*, a gene encoding a phosphatase suppressing mTOR signaling activity, have been identified in individuals with macrocephaly and ASD.^49^^,^^50^ Deletion of *Pten* in the forebrain of mouse models results in macrocephaly, seizures, and abnormal social interactions.^51^ *Pten*-null neurons exhibited increased neuronal soma size, dendritic length, and number of dendritic spines.^51^ Notably, rapamycin, an mTORC1 inhibitor, reversed the neuronal phenotypes, resulting in the amelioration of behavioral abnormalities in *Pten*-KO mice.^52^ These findings suggest that the mTOR pathway is crucial for *PTEN*-associated abnormal phenotypes. Similarly, MeCP2 gain- and loss-of-function have been reported to affect mTOR signaling. Analyses of postmortem brain tissues from RTT patients with *MECP2* mutations revealed disruption of the nucleolin-mTOR-P70S6K signaling cascade.^30^ Dysregulated mTOR phosphorylation was associated with reduced activation of downstream effectors, such as P70S6K, and mislocalization of nucleolin from the nucleolus to the cytoplasm.^30^ In addition, impaired mTOR signaling and downregulation of translation initiation factors were observed in RTT cortex,^53^ suggesting that *MECP2* mutations impair mTOR-dependent translation in the human brain. Several genes involved in the mTOR pathway are dysregulated in MeCP2-overexpressing mice, leading to enhanced mTOR signaling.^1^ A previous study demonstrated that the phosphorylation levels of S6 kinase (S6K), an index of mTORC1 signaling activation, were significantly higher in the cortical tissues of MeCP2-overexpressing mice than in those of control mice.^43^ The present study confirmed that the phosphorylation levels of ribosomal protein S6 are increased in MDS model neurons, including human iPSC-derived neurons. Notably, downregulating miR-199a expression suppressed the aberrant enhancement of the mTOR activity in MDS model neurons both *in vitro* and *in vivo*. Our results suggest that miR-199a contributes to the pathophysiology of MDS by regulating mTOR activity.

We also demonstrated that inhibition of miR-199a-5p mainly downregulated neuronal soma size and mTOR signaling activity, whereas blocking miR-199a-3p had lesser effects. In contrast, the dendritic outgrowth in MDS neurons was markedly decreased by miR-199a-3p inhibition. Thus, the two mature forms of miR-199a, miR-199a-5p and -3p, may have distinct effects on the phenotype of MDS neurons through interactions with different target genes. We previously reported that miR-199a-5p upregulates mTOR signaling activity by repressing the expression of mTOR signaling inhibitors, including Sirtuin-1, hypoxia-inducible factor 1 subunit alpha, and *Pde4d*.^8^ In the present study, we further confirmed that *PDE4D* functions as a downstream target of miR-199a-5p in MDS model neurons, supporting its involvement in mTOR pathway dysregulation in this context. *PDE4D* encodes a phosphodiesterase that hydrolyzes cAMP, and dysregulation of this signaling cascade can modulate the interaction between Rheb and mTOR, thereby influencing mTOR pathway activity.^37^ Moreover, we identified *QKI* as a potential downstream target of miR-199a-3p. In our previous study, we demonstrated that *QKI* knockdown in primary neurons led to impaired dendritic growth, whereas its overexpression promoted dendritic development, highlighting its critical role in neurite morphogenesis.^38^ In addition to *QKI*, previous reports have suggested that miR-199a-3p can target *Smad1*, a transcription factor involved in bone morphogenetic protein and transforming growth factor β signaling.^27^^,^^54^ Smad1 is a key downstream signaling molecule in the transforming growth factor β (TGF-β) pathway. Additionally, Smad-dependent TGF-β signaling negatively regulates neurite outgrowth in neurons.^55^ We previously demonstrated that overexpression of *Smad1* inhibits neurite growth, whereas knockdown of *Smad1* enhances neurite development.^55^ These results indicate the possibility that miR-199a-5p and -3p contribute to distinct pathological features in MDS neurons through different molecular pathways. Furthermore, spatiotemporal analyses of miR-199a expression using high-throughput omics technologies, such as single-cell and spatial transcriptomics, may provide novel insights into its broader role in neurodevelopmental features of MDS beyond neuronal morphology.

In our cortical organoid model derived from MDS patient iPSCs, two-photon Ca^2+^ imaging revealed a reduction in neuronal activity, suggesting decreased cortical network activity in MDS organoids. Inhibition of miR-199a-5p partially rescued this impaired activity, whereas inhibition of miR-199a-3p had no apparent effect. This finding contrasts with our *in vitro* observations of enhanced dendritic outgrowth in MDS neurons and a previous report showing increased dendritic complexity, increased number of excitatory synapses, and elevated synchronized bursts of MDS neurons.^56^ These findings suggest the presence of more active synapses on MDS neurons; however, MDS neurons exhibited more immature and less mature spines compared with NC neurons.^56^ The spine density in MDS neurons increased during early neurogenesis but decreased with age.^43^ Interestingly, a study in *Mecp2*-overexpressing mouse models reported a biphasic course of neuronal function, in which early phases (up to ∼20 weeks of age) were characterized by features of hyperexcitability, such as aggressive behavior and enhanced learning ability, whereas later phases (beyond ∼30 weeks) exhibited hypoactivity, motor incoordination, and premature death.^18^ These progressive neuro-behavioral declines in older transgenic mice were accompanied by epileptic activity.^18^ Excitotoxicity caused by excessive release of glutamate during seizures results in decrease in dendritic spine density.^57^ From a clinical perspective, developmental and intellectual regressions have been reported among approximately 40% of individuals with MDS. The onset of regression has been observed in parallel with seizure occurrence or exacerbation.^16^^,^^58^ This raises the possibility that the reduced neuronal activity observed in our cortical organoids may represent a later-stage phenotype resulting from early hyperexcitability and subsequent excitotoxic cell death. Supporting this notion, a study using MeCP2 triplication mice demonstrated that elevated MeCP2 expression increases glutamate levels, thereby inducing excitotoxicity and neuronal degeneration.^59^ Thus, further studies with detailed functional analyses are warranted to clarify molecular and cellular mechanisms underlying the progressive neurological dysfunction observed in human MDS.

In conclusion, MeCP2 overexpression resulted in pathological neuronal phenotypes, including increased dendritic outgrowth, overgrowth of neuronal soma, enhanced mTOR signaling activity, a high density of excitatory synapses, and altered neuronal network activity, which were mediated by an increase in miR-199a, acting downstream of MeCP2. Taken together, this study provides evidence for the structural and molecular basis of neuronal dysfunction in the pathophysiology of MDS. These findings suggest that targeting the MeCP2/miR-199a axis may represent a novel therapeutic strategy for MDS. However, efficient and specific delivery of miRNA inhibitors to the CNS remains a major challenge. Recent advances in AAV-mediated delivery and chemically modified antisense oligonucleotides may help address this issue and warrant further investigation.

### Limitations of the study

This study has several limitations. First, the models used, including mouse models and patient-derived iPSCs, do not fully replicate the complexity of the human CNS. While informative, these models may not reflect transcriptomic abnormalities observed in postmortem RTT brains^60^^,^^61^^,^^62^^,^^63^ or the clinical heterogeneity seen in MDS patients.^16^^,^^58^ Moreover, the limited number of iPSC lines may not fully account for inter-patient variability. To improve reproducibility and generalizability, future studies will include additional patient-derived lines with diverse duplication sizes and clinical features. In some experiments, the number of animals was also limited, which may affect the generalizability of the findings. Second, although this study identified distinct downstream targets of miR-199a-5p and miR-199a-3p, such as *PDE4D* and *QKI*, further investigation is needed to fully delineate the broader molecular networks regulated by these miRNAs. Third, while this study included functional assessments using brain organoids, comprehensive evaluation of neuronal network function and *in vivo* behavioral consequences remains to be explored in future studies.

## Resource availability

### Lead contact

Requests for additional information, resources, and reagents should be directed to and will be fulfilled by the lead contact, Satoru Takahashi (satoru5p@asahikawa-med.ac.jp).

### Materials availability

Materials generated in this study are available upon request, subject to a completed Materials Transfer Agreement.

### Data and code availability

• All data reported in this study will be shared by the lead contact upon request. The uncropped western blot images supporting Figures 9 and S1 are provided as Supplementary Data (Data S1).
• This study does not report any code.
• Any additional information required to reanalyze the data reported in this study is available from the lead contact upon request.

## Acknowledgments

We appreciate the technical assistance provided by the Animal Center at Nagoya University. We would like to express our sincere appreciation to all our coworkers and collaborators for this research, as well as to members of the MECP2 Duplication Japan (Family Association) for their help and participation in establishing disease-specific iPS cells. This work was supported by the Japan Agency for Medical Research and Development (AMED) (JP21ek0109411 to K.T.; JP22ek0109498 and JP23ek0109498 to S.T. and K.T.); the 10.13039/501100001691Japan Society for the Promotion of Science (10.13039/501100001691JSPS) 10.13039/501100001691KAKENHI (JP21K06834, JP19H05211, and JP18K06484 to K.T.; JP25K02553 and JP22H02771 to F.O.); and a grant from the 10.13039/100012045Kawano Masanori Memorial Public Interest Incorporated Foundation for the Promotion of Pediatrics (29-11). This work was also partially supported by the Npo Rett Syndrome Support Organization (2019-01-09 and 2020-01-27 to K.T.) and by RIKEN-Nagoya University Internal grants (to K.T. and Y.H.).

## Author contributions

Y.Akaba. and K.T. performed the experiments. K.T., H.N., and K.N. designed the miR-199a sponge plasmid vectors. K.S. designed the gRNAs used for generating *MIR199A2*-knockout MDS iPSC line. R.K., P.F.C., Y.S., Y.H., I.K., D.M., Y.Arioka., H.O., A.N., S.M., and N.O. generated patient-derived iPSCs. T.Y. performed array-CGH to define duplication sizes. S.A., M.N., and F.O. generated *MIR199A2*-knockout MDS iPSC lines, generated neural organoids, and performed two-photon calcium imaging. Y.Akaba., S.T., F.O., and K.T. wrote the manuscript. K.T. and S.T. designed and supervised the study. All authors reviewed and approved the final manuscript.

## Declaration of interests

The authors declare that this study was conducted in the absence of any commercial or financial relationships that could be construed as potential conflicts of interest.

## STAR★Methods

### Key resources table

**Table:** 

REAGENT or RESOURCE	SOURCE	IDENTIFIER
**Antibodies**		

goat anti-SOX17	R&D Systems	Cat#AF1924
mouse anti-αSMA	R&D Systems	Cat#MAB1420
mouse anti-αSMA	Sigma-Aldrich	Cat#A2547
mouse anti-βIII-tubulin	R&D Systems	Cat#MAB1195
mouse anti-βIII-tubulin	Sigma-Aldrich	Cat#T8660
guinea pig anti-MAP2	Synaptic Systems	Cat#188004
mouse anti-NeuN	Merck Millipore	Cat#MAB377
rabbit-anti-VGLUT1	Synaptic Systems	Cat#135302
mouse anti-PSD95	Thermo Fisher Scientific	Cat#MA1-046
rabbit anti-S6	Cell Signaling Technology	Cat#2217
mouse anti-S6	Proteintech	Cat#66886-1-Ig
rabbit anti-p-S6 Ser235/236	Cell Signaling Technology	Cat#4858
rabbit anti-mTOR	Cell Signaling Technology	Cat#2983
mouse anti-mTOR	Proteintech	Cat#66888-1-Ig
rabbit p-mTOR Ser2448	Cell Signaling Technology	Cat#5536
CF647 donkey anti-guinea pig IgG	Biotium	Cat#20041-1
Alexa Fluor 555 donkey anti-rabbit IgG	Invitrogen	Cat#A32794
Alexa Fluor 488 donkey anti-mouse IgG	Invitrogen	Cat#A21202
Alexa Fluor 488 donkey anti-goat IgG	Abcam	Cat#ab150129
Alexa Fluor 594 donkey anti-mouse IgG	Abcam	Cat#ab150108
Alexa Fluor Plus 555 goat anti-mouse IgG	Invitrogen	Cat#A32727
Alexa Fluor Plus 555 donkey anti-goat IgG	Invitrogen	Cat#A32816
Alexa Fluor 555 donkey anti-mouse IgG	Invitrogen	Cat#A31570
anti-rabbit IgG, HRP-linked whole antibody from donkey	GE Healthcare	Cat#NA934V

**Bacterial and virus strains**		

Lentiviruses	This paper	N/A
AAV-DJ-hSyn-jGCaMP7f	This paper	N/A

**Chemicals, peptides, and recombinant proteins**		

DNase I	Sigma-Aldrich	Cat#D4527
Papain	Sigma-Aldrich	Cat#P3125
Poly-L-lysine	Sigma-Aldrich	Cat#P2636
Neurobasal medium	Invitrogen	Cat#21103049
Neurobasal medium	Sigma-Aldrich	Cat#10888022
B27 supplement	Invitrogen	Cat#17504044
B27 supplement without vitamin A	Invitrogen	Cat#A3353501
GlutaMAX	Invitrogen	Cat#35050061
Cytosine β-D-arabinofuranoside	Sigma-Aldrich	Cat#C1768
SB431542	Cayman	Cat#13031
CHIR99021	Cayman	Cat#13122
Dorsomorphin	Sigma-Aldrich	Cat#P5499
TrypLE select	Gibco	Cat#12563-011
DMEM/F12	Gibco	Cat#10565018
N2 supplement	Gibco	Cat#17502048
N2 supplement	Wako	Cat#141-08941
40% Glucose	Sigma-Aldrich	Cat#G8769
HEPES	Sigma-Aldrich	Cat#H0887
bFGF	Wako	Cat#064-04541
bFGF	Peprotech	Cat#100-25
hLIF	Nacalai	Cat# NU0013-1
Y27632	Cayman	Cat#10005583
Y27632	MedChemExpress	Cat#HY-10583
FGF8	Peprotech	Cat#100-25
Purmorphamine	Cayman	Cat#10009634
Poly-L-ornithine	Sigma-Aldrich	Cat#P3655
Laminin	Gibco	Cat#23017-015
Fibronectin	Wako	Cat#063-05591
DAPT	Cayman	Cat#13197
BDNF	Peprotech	Cat#450-02
GDNF	R&D Systems	Cat#212-GD-010
Ascorbic acid	Sigma-Aldrich	Cat#A4544
TGF-β3	R&D Systems	Cat#243-B3-002
dbcAMP	Sigma-Aldrich	Cat#D0627
KSR	Thermo Fisher Scientific	Cat#10828028
MEM-NEAA	Wako	Cat#139-15651
L-glutamine	Wako	Cat#073-05391
β-mercaptoethanol	Wako	Cat#133-14571
LDN-193189	Sigma-Aldrich	Cat#SML0559
A-83-01	Wako	Cat#035-24113
IWR-1-*endo*	Wako	Cat#037-25131
Penicillin-Streptomycin solution	Wako	Cat#168-23191
iMatrix-511	Nippi	Cat#892021
Opti-MEM	Thermo Fisher Scientific	Cat#31985070
Lipofectamine STEM	Thermo Fisher Scientific	Cat#STEM00008
AK02N medium	Ajinomoto	Cat#RCAK02N
Accutase	Nacalai	Cat#12679-54
Ex Taq Buffer	Takara Bio	Cat#RR001A
Proteinase K	Wako	Cat#161-28701
PrimeSTAR Max DNA Polymerase	Takara Bio	Cat#R045A
Polyethyleneimine	Polyscience	Cat#24765-1
OptiPrep	Alere Technologies	Cat#AXS-1114542
Triton X-100	Sigma-Aldrich	Cat#X100RS
NP40 buffer	Invitrogen	Cat#FNN0021
Protease inhibitor mixture	Nacalai	Cat#03969-21
Phosphatase inhibitor mixture	Nacalai	Cat#07575-51
Blocking One	Nacalai	Cat#03953-95
ECL Prime Western Blotting Detection Reagent	GE Healthcare	Cat#RPN2232

**Critical commercial assays**		

mirVana miRNA Isolation Kit	Ambion	Cat#AM1560
SuperScript VILO cDNA synthesis kit	Invitrogen	Cat#11756050
TaqMan Gene Expression Assays	Applied Biosystems	Various
TaqMan MicroRNA Reverse Transcription Kit	Applied Biosystems	Cat#4366596
TaqMan MicroRNA Assay Kit	Applied Biosystems	Various

**Deposited data**		

aCGH data of patient 1	ClinVar	Submission ID: SUB14765916
aCGH data of patient 2	ClinVar	Submission ID: SUB14765957

**Experimental models: Cell lines**		

HEK293T	ATCC	CRL-3216
iPSC line derived from patient 1	This paper	AC1140206AS
iPSC line derived from patient 2	This paper	HiPS-AC8783
Control iPSC line 1	RIKEN BRC	201B7 (HPS0063)
Control iPSC line 2	RIKEN BRC	1383D6 (HPS1006)

**Experimental models: Organisms/strains**		

MeCP2 Tg1 mice	The Jackson Laboratory	RRID:IMSR_JAX:008679
*miR-199a-2*-KO mice	Tsujimura et al.^8^	N/A
C57BL/6J wild-type littermate mice	This paper	N/A

**Recombinant DNA**		

pCMV-VSV-G-RSV-Rev	Tsujimura et al.^8^	N/A
pCAG-HIVgp	Tsujimura et al.^8^	N/A
pLV-sh-*Mecp2*-1 (GCTGGAAAGTATGATGTAT ATCTCGAGATATACATCATACTTTCCAGC)	VectorBuilder	VB210627-1040ecd
pLV-sh-*Mecp2*-2 (TGACAAAGCTTCCCGATTA ACCTCGAGGTTAATCGGGAAGCTTTGTCA)	VectorBuilder	VB210627-1032rmk
pLV-sh-*Mecp2*-3 (ACCACCTAAGAAGCCCAAA TCCTCGAGGATTTGGGCTTCTTAGGTGGT)	VectorBuilder	VB210627-1035yfj
pLV-sh-*Mecp2*-4 (CTGGGAAGTATGATGTGTA TTCTCGAGAATACACATCATACTTCCCAG)	VectorBuilder	VB210627-1037nua
pLV-sh-Scramble (CCTAAGGTTAAGTCGCCCT CGCTCGAGCGAGGGCGACTTAACCTTAGG)	VectorBuilder	VB010000-0001mty
pLV-sponge-miR-199a-5pi	Tsujimura et al.^8^	N/A
pLV-sponge-miR-199a-3pi	Tsujimura et al.^8^	N/A
pHelper	Cat#A3353501	VPK-400-DJ
pAAV2/DJ rep/cap	Cell Biolabs	VPK-400-DJ
pAAV-hSyn-jGCaMP7f	Addgene	104488
pRP-2gRNA-2-mCherry-U6-MIR199A2-gRNA1-U6-MIR199A2-gRNA2 (gRNA1: GAGCAACGCCATGGA CCGCTGTTTTAGAGCTAGAAATAGCAAGTTAAAAT AAGGCTAGTCCGTTATCAACTTGAAAAAGTGGCA CCGAGTCGGTGC, gRNA2: GAAATCCTCTCCCG GTCCTCGTTTTAGAGCTAGAAATAGCAAGTTAAA ATAAGGCTAGTCCGTTATCAACTTGAAAAAGTG GCACCGAGTCGGTGC)	VectorBuilder	VB250212-1216vhj
pCAG-1BPNLS-SpCas9-1BPNLS-2A-GFP	Addgene	87109

**Software and algorithms**		

FIJI (ImageJ)	NIH	RRID:SCR_002285
MATLAB	MathWorks	RRID:SCR_001622
GraphPad Prism 10	GraphPad Software	RRID:SCR_002798

### Experimental model and study participant details

#### Mice

MeCP2 Tg1 mice, which express MeCP2 at twice the wild-type levels in the brain, were originally generated in Dr. Huda Zoghbi’s laboratory and purchased from The Jackson Laboratory.^18^ Male Tg1 mice were crossed with female C57BL/6J WT mice and maintained on the C57BL/6J genetic background. *miR-199a-2*-KO mice were previously generated and maintained on a C57BL/6J genetic background.^8^ Mice were housed in a temperature-controlled environment under a 12-hour light/dark cycle with *ad libitum* access to food and water. All aspects of animal care and treatment were conducted in accordance with the guidelines of the Experimental Animal Care Committee of Nagoya University. All experiments were conducted using male mice, including Tg1, Tg1; *miR-199a-2*^+/-^, and age-matched WT littermates, to avoid potential sex-related variability.

#### Generation of iPSC from patients with MDS

As previously reported, iPSCs were generated from peripheral blood mononuclear cells obtained from two patients with MDS using episomal vectors (pCE-hOCT3/4, pCE-hSK, pCE-hUL, pCE-mp53DD, and pCXB-EBNA1).^64^ The detailed phenotypes of the two patients with MDS were compiled in Table S1. Patient 1 (AC1140206AS), referred to as MDS-1, was a 13-year-old Japanese male with severely delayed developmental milestones. He was able to walk unaided at two and a half years old, but he never acquired speech sounds. Comparative genomic hybridization (CGH) associated with single-nucleotide polymorphism (SNP) analysis was performed using an Agilent CGH + SNP 180 K array (Agilent Technologies, Santa Clara, CA, USA) and showed an ∼434 kb duplication at Xq28 (arr[GRCh37] Xq28(153,168,032_153,601,836)x2) encompassing 15 genes including the *IRAK1, MECP2* and *FLNA* (Figure S2A, ClinVar submission ID: SUB14765916). Blood sampling for iPSC generation was performed when the patient was 7 years old. Patient 2 (HiPS-AC8783), referred to as MDS-2, was a 14-year-old Japanese male who had not acquired meaningful words and attended a school for children with special needs, relying on a wheelchair. He experienced recurrent vomiting and respiratory infections from early infancy and began having epileptic seizures at 6 years old. CGH showed an ∼1.8 Mb deletion in the pseudoautosomal region at Xp22.33 (arr[GRCh37] Xp22.33(60701_1,851,679)x1) encompassing 14 genes including the *SHOX* and *SLC25A6* and an ∼2.4 Mb duplication at Xq28 (arr[GRCh37] Xq28(152,788,477_155,226,944)x2) encompassing 71 genes including the *L1CAM* and *MECP2* (Figure S2B, ClinVar submission ID: SUB14765957). All iPSC lines from MDS patients (MDS-1 and MDS-2) were derived from male individuals. All the generated iPSCs had the ability to differentiate into the three germ layers *in vitro* (Figure S2C). The human iPSC line 201B7 (HPS0063)^65^ and 1383D6 (HPS1006),^66^ referred to as NC-1 and NC-2, respectively, were used as normal controls. These lines were provided by the RIKEN BioResource Research Center (Tsukuba, Japan). iPSCs were cultured with or without feeder cells, as previously described.^67^ The study protocol was reviewed and approved by the Research Ethics Committees of Nagoya University, Asahikawa Medical University, Kyusyu University, and Aichi Developmental Disability Center.

### Method details

#### Primary hippocampal neuron culture

Primary hippocampal neurons were collected using an established method.^68^ Briefly, hippocampi were dissected from embryonic day 17.5 (E17.5) mice and dissociated by trituration with 60 mg/ml DNase I (Sigma-Aldrich, Burlington, MA, USA) after pre-incubation with 0.1% papain (Sigma-Aldrich). The cells were plated on poly-L-lysine (Sigma-Aldrich)-coated culture dishes at densities of 2.0 × 10^4^ cells/cm^2^ and 5.0 × 10^4^ cells/cm^2^ for immunocytochemistry and RNA extraction, respectively. Neurons were maintained in a serum-free neuronal culture medium consisting of neurobasal medium (Invitrogen, Carlsbad, CA, USA) supplemented with B27 (Invitrogen) and 0.5 mM GlutaMAX (Invitrogen). Cytosine β-D-arabinofuranoside (Sigma-Aldrich) was added once to eliminate proliferating glial cells. Half of the medium was replaced with fresh culture medium every 3 days.

#### Neuronal differentiation

*In vitro* neuronal induction was performed as previously described^69^ with slight modifications. iPSCs were pretreated with 3 μM SB431542 (Cayman, Ann Arbor, MI, USA), 3 μM CHIR99021 (Cayman), and 3 μM dorsomorphin (Sigma-Aldrich) for 1 week. They were then dissociated by TrypLE select (Gibco, Billings, MT, USA) and cultured in neurosphere medium consisting of MHM medium (DMEM/F12 [Gibco] plus N2 supplement [Gibco], 0.6% glucose [Sigma-Aldrich], and 5 mM HEPES [Sigma-Aldrich]) supplemented with B27, 20 ng/ml bFGF (Wako, Osaka, Japan), 10 ng/ml hLIF (Nacalai, Kyoto, Japan), 10 μM Y27632 (Cayman), 3 μM CHIR99021, 2 μM SB431542, 100 ng/ml FGF8 (Peprotech, Cranbury, NJ, USA), and 1 μM purmorphamine (Cayman). After 2 weeks, neurospheres were dissociated and plated on poly-L-ornithine (Sigma-Aldrich) / laminin (Gibco) / fibronectin (Wako)-coated dishes at densities of 3.0 × 10^4^ cells/cm^2^and 5.0 × 10^4^ cells/cm^2^ for immunocytochemistry and RNA extraction, respectively. Cells were maintained in dopaminergic neuron medium consisting of MHM medium supplemented with B27, 10 μM DAPT (Cayman), 20 ng/ml BDNF (Peprotech), 20 ng/ml GDNF (R&D Systems, Minneapolis, MN, USA), 0.2 mM ascorbic acid (Sigma-Aldrich), 1 ng/ml TGF-β3 (R&D Systems), and 0.5 mM dbcAMP (Sigma-Aldrich). Each biological replicate (n) represents an independent neuronal differentiation from the same iPSC line.

#### Generation of neural organoids from human iPSCs

Cortical organoids were generated from human iPSCs as previously described.^40^ Briefly, single-cell suspensions of undifferentiated hiPSCs were plated into low-adherent 96-well V-bottom plates (1.0 × 10^4^ cells/well) in DMEM/F12 (Sigma-Aldrich) supplemented with 15% KnockOut Serum Replacement (KSR) (Thermo Fisher Scientific, Waltham, MA, USA), 1% Minimum Essential Medium Non-Essential Amino Acid (MEM-NEAA) (Wako), 1% L-glutamine (Wako), 100 μM β-mercaptoethanol (Wako), 100 nM LDN-193189 (Sigma-Aldrich), 500 nM A-83-01 (Wako), 2 μM IWR-1-*endo* (AdooQ BioScience, Irvine, CA, USA), and 50 μM Y27632 (MedChemExpress, Monmouth Junction, NJ, USA). Half of the medium was replaced with fresh medium every other day. Y27632 was removed on day 4. On day 10, the organoids were transferred to spinning culture (70 rpm) in low-adherent 6-cm dishes and maintained in a 1:1 mixture of DMEM/F12 and Neurobasal medium (Thermo Fisher Scientific) supplemented with 0.5% N2 supplement (Wako), 1% B27 supplement without vitamin A (Invitrogen), 0.5% MEM-NEAA, 1% L-glutamine, 0.025% insulin (Wako), 50 μM β-mercaptoethanol, and 1% penicillin/streptomycin (Wako), and 20 ng/mL bFGF (Peprotech). The medium was changed every other day until day 18. From day 18 onward, organoids were maintained in a 1:1 mixture of DMEM/F12 and Neurobasal medium supplemented with 20 ng/mL BDNF (Peprotech) and 200 μM ascorbic acid (Sigma-Aldrich), with medium changes every other day thereafter. The generated organoids were infected with AAVDJ-hSyn-jGCaMP7f on day 40 and subjected to two-photon calcium imaging on day 50. Alternatively, the organoids were infected with lentivirus expressing sponge-miR-199a-5p or sponge-miR-199a-3p on day 20 and with AAVDJ-hSyn-jGCaMP7f on day 70 and subjected to two-photon calcium imaging on day 80.

#### Generation of *MIR199A2*-knockout MDS iPSC line

An isogenic human MDS iPSC line in which the *MIR199A2* locus was homozygously knocked out was generated using CRISPR-Cas9-mediated genome editing. Briefly, MDS iPSCs were dissociated into single cells and plated onto iMatrix-511 (Nippi, Tokyo, Japan)-coated 60 mm dish. Three days after plating, 5 μg of pCAG-1BPNLS-SpCas9-1BPNLS-2A-GFP and 5 μg of pRP-2gRNA-2-mCherry-U6-*MIR199A2*-gRNA1-U6-*MIR199A2*-gRNA in 100 μl Opti-MEM (Thermo Fisher Scientific) were mixed with 8 μl Lipofectamine STEM (Thermo Fisher Scientific) in 100 μl Opti-MEM for 15 min and then applied to iPSCs in a dropwise manner. The cells were incubated for 6 h, washed with AK02N medium (Ajinomoto, Tokyo, Japan), and then incubated in AK02N medium supplemented with 10 μM Y27632 (MedChemExpress). At 24 h post-transfection, the transfected cells were dissociated into single cells with accutase (Nacalai), passed through cell strainers, and subjected to a cell sorter (CytoFLEX SRT; Beckman Coulter, Brea, CA, USA). GFP- and mCherry-double-positive cells were sorted into 10 ml of AK02N medium containing 20 μM Y-27632 and 1% penicillin/streptomycin for 1 h. The sorted cells were resuspended with 3 ml of AK02N medium containing 20 μM Y-27632 and 1% penicillin/streptomycin and seeded onto iMatrix-511-coated 60 mm dishes. After 48 h, the media were changed to fresh ones with 10 μM Y-27632. Five days after cell sorting, the cells were dissociated into single cells with accutase and plated onto iMatrix-511-coated 60 mm dish. Individual colonies were manually picked up, seeded on iMatrix-511-coated 48-well plates dish, and cultured until confluency. Genomic DNA was extracted using Ex Taq Buffer (Takara Bio, Shiga, Japan) containing Proteinase K (Wako). Target loci were amplified by PCR using PrimeSTAR Max DNA Polymerase (Takara Bio). Purified PCR amplicons were sequenced and indel profiles were analyzed using SnapGene (GSL Biotech, Chicago, IL, USA). Five clones were positive for the *MIR199A2*-KO lines, and three independent KO clones were used for subsequent analyses.

#### Constructs

Lentiviral vectors expressing Scramble short-hairpin RNA (shRNA) [pLV-U6>Scramble shRNA#1], sh-*Mecp2*-1 [pLV-U6>sh-m*Mecp2* shRNA#1], sh-*Mecp2*-2 [pLV-U6>sh-h*MECP2* shRNA#1, sh-m*Mecp2* shRNA#2/3], sh-*Mecp2*-3 [pLV-U6>sh-h*MECP2* shRNA#5, sh-m*Mecp2* shRNA#4], and sh-*MECP2*-4 [pLV-U6>sh-h*MECP2* shRNA#3] were purchased from VectorBuilder (Chicago, IL, USA). sh-*Mecp2*-1 targets only mouse *Mecp2,* while sh-*MECP2*-4 targets only human *MECP*2. sh-*Mecp2*-2 and -3 target both mouse *Mecp2* and human *MECP2*. A non-targeting shRNA (sh-Scramble) was used as a negative control. The sponges against miR-199a-5p- or miR-199a-3p-expressing lentivirus plasmids were constructed by inserting the following oligonucleotides into the HpaI and XhoI sites of pLLX: sponge-miR-199a-5p-Fw, 5′- gacgttaacGAACAGGTACGTCTAACACTGGGGAACAGGTACGTCTAACACTGGGGAACAGGTACGTCTAACACTGGGttttttctcgaggtc-3′, and sponge-miR-199a–5p-Rv, 5′-gacctcgagaaaaaaCCCAGTGTTAGACGTACCTGTTCCCCAGTGTTAGACGTACCTGTTCCCCAGTGTTAGACGTACCTGTTCgttaacgtc-3’; sponge-miR-199a-3p-Fw, 5′-gacgttaacTAACCAATGCATCGACTACTATTAACCAATGCATCGACTACTATTAACCAATGCATCGACTACTATttttttctcgaggtc-3′, and sponge-miR-199a-3p-Rv, 5′- gacctcgagaaaaaaATAGTAGTCGATGCATTGGTTAATAGTAGTCGATGCATTGGTTAATAGTAGTCGATGCATTGGTTAgttaacgtc-3’. For CRISPR-Cas9-mediated knockout of *MIR199A2*, a dual gRNA system targeting the *MIR199A2* locus was used. The target sequences for gRNA#1 and gRNA#2 were 5′-GAGCAACGCCATGGACCGCT-3′ and 5′- GAAATCCTCTCCCGGTCCTC-3′, respectively. The gRNAs and SpCas9 were expressed from separate plasmids; SpCas9 was co-expressed with GFP, and the gRNA plasmid with mCherry to facilitate FACS-based selection of transfected cells.

#### Lentivirus production

Lentiviruses were produced as previously described.^8^ Briefly, HEK293T cells were co-transfected with the lentiviral plasmids pCMV-VSV-G-RSV-Rev and pCAG-HIVgp using polyethyleneimine (Polyscience, Warrington, PA, USA). After 48h, culture supernatants were collected, centrifuged at 6,000 × *g* for 12 h, and the pellet was resuspended in culture medium. The viral titer was determined by qPCR and typically ranged from 3.5 × 10^8^ to 5.2 × 10^8^ TU/mL. The virus was applied to primary hippocampal neurons and iPSC-derived neurons one day after plating.

#### AAV production

Adeno-associated virus (AAV) vectors were generated in HEK293T cells as previously described.^70^ Briefly, AAVDJ-hSyn-jGCaMP7f was produced by transfecting HEK293T cells with pHelper, the AAV2/DJ rep/cap plasmid, and the genomic plasmid pAAV-hSyn-jGCaMP7f. Transfected cells were harvested 3 days after transfection and lysed by freeze–thaw cycles. After centrifugation, the supernatant was loaded onto iodixanol gradients (15%, 25%, 40%, and 58% of OptiPrep (Alere Technologies, Jena, Germany). Following centrifugation at 16,000 × *g* for 4 h at 4°C, the 40% iodixanol fraction was collected and used for infection experiments. AAV genomic titers were quantified by qPCR and ranged from 4.1 × 10^12^ to 5.5 × 10^12^ viral genomes/mL.

#### Immunocytochemistry

Cells were fixed in 4% paraformaldehyde (PFA) for 20 min, washed with phosphate-buffered saline (PBS), permeabilized, and blocked with blocking buffer (3% fetal bovine serum (FBS) and 0.1% Triton X-100 (Sigma-Aldrich) in PBS) at room temperature (RT). The cells were then incubated with primary antibodies diluted in blocking buffer overnight at 4°C. The primary antibodies were rinsed off, and the corresponding secondary antibodies were added for 3 h at RT. After washing with PBS, the cells were mounted on glass slides. The images were captured using an all-in-one fluorescence microscope (BZ-X800, Keyence, Osaka, Japan).

#### Tissue preparation and immunohistochemistry

Experimental mice were anesthetized with an intraperitoneal injection of a mixture containing medetomidine, midazolam, and butorphanol. They were then transcardially perfused with cold PBS, followed by 4% PFA. The brain samples were postfixed with 4% PFA for 6 h and subsequently immersed in 30% sucrose in PBS at 4°C. Tissue sections (40 μm thickness) were prepared with a cryostat microtome (CM1950, Leica Biosystems, Wetzlar, Germany). The sections were permeabilized and blocked with blocking buffer at RT. They were then incubated with primary antibodies overnight at 4°C. After washing with PBS, the sections were incubated with secondary antibodies for 4 h at RT. Finally, the sections were washed with PBS and mounted on glass slides. Fluorescence images were acquired using a confocal laser scanning microscope (FV1000-D; Olympus, Tokyo, Japan). A Z series of 20 images was collected with 1-μm steps and stacked using ImageJ software.

#### Western blotting

The cell extracts were isolated by first washing the cells with cold PBS followed by lysis with NP40 buffer (Invitrogen) containing 1% protease inhibitor mixture (Nacalai) and 1% phosphatase inhibitor mixture (Nacalai). Lysates were centrifuged at 20,000 x *g* for 15 min. Total cell lysates were subjected to SDS-PAGE and transferred to a PVDF membrane (GE Healthcare, Chicago, IL, USA) using Trans-Blot Turbo transfer system (Bio-Rad, Hercules, CA, USA). The membrane was blocked with Blocking One (Nacalai) and incubated with a primary antibody solution overnight at 4°C. Then, the membrane was incubated with a peroxidase-conjugated secondary antibody solution at RT for 3 h. Immunoreactive bands were detected by enhanced chemiluminescence using ECL Prime Western Blotting Detection Reagent (GE Healthcare). The intensities of the bands were densitometrically quantified using ImageJ software.

#### Antibodies

The following primary antibodies were used for immunocytochemistry and immunohistochemistry: goat anti-SRY-box transcription factor 17 (SOX17) (1:100; R&D Systems), mouse anti-alpha-smooth muscle actin (αSMA) (1:500; Sigma-Aldrich), mouse anti-αSMA (1:100; R&D Systems), mouse anti-βIII-tubulin (1:1000; Sigma-Aldrich), mouse anti-βIII-tubulin (1:100; R&D Systems), guinea pig anti-microtubule-associated protein 2 (MAP2) (1:1000; Synaptic Systems, Goettingen, Germany), rabbit anti-phospho-S6 ribosomal protein (p-S6) Ser235/236 (1:1000 or 1:500; Cell Signaling Technology, Danvers, MA, USA), mouse anti-S6 ribosomal protein (1:1000; Proteintech, Rosemont, IL, USA), rabbit anti-phospho-mTOR (p-mTOR) Ser2448 (1:1000; Cell Signaling Technology), mouse anti-mTOR (1:1000; Proteintech), mouse anti-neuronal nuclei (NeuN) (1:500; Merck Millipore, Darmstadt, Germany), rabbit anti-vesicular glutamate transporter 1 (VGLUT1) (1:500; Synaptic Systems), and mouse anti-postsynaptic density protein 95 (PSD95) (1:500; Thermo Fisher Scientific). The secondary antibodies used included: CF647 donkey anti-guinea pig IgG (H + L) (1:1000; Biotium, Fremont, CA, USA), Alexa Fluor 555 donkey anti-rabbit IgG (H + L) (1:1000 or 1:500; Invitrogen, Carlsbad, CA, USA), Alexa Fluor 488 donkey anti-mouse IgG (H + L) (1;500; Invitrogen), Alexa Fluor 488 donkey anti-goat IgG (H + L) (1;2000; Abcam, Cambridge, UK), Alexa Fluor 594 donkey anti-mouse IgG (H + L) (1;2000; Abcam), Alexa Fluor Plus 555 donkey anti-goat IgG (H+L) (1:400; Invitrogen), Alexa Fluor 555 donkey anti-mouse IgG (H+L) (1:400; Invitrogen), and Alexa fluor 555 goat anti-mouse IgG (H + L) (1:500; Invitrogen). The nuclei were stained with Hoechst 33258 (1:1000; Wako). For Western blotting, the following primary antibodies were used: rabbit anti-S6 ribosomal protein (1:500; Cell Signaling Technology), rabbit anti-phospho-S6 ribosomal protein (p-S6) Ser235/236 (1:500; Cell Signaling Technology), rabbit anti-mTOR (1:500; Cell Signaling Technology), and rabbit anti-phospho-mTOR (p-mTOR) Ser2448 (1:500; Cell Signaling Technology). The following secondary antibody was used: anti-rabbit IgG, HRP-linked whole antibody from donkey (1:10000; GE Healthcare).

#### Morphometric analysis of cultured neurons

Neuronal soma size and dendritic outgrowth *in vitro* were analyzed as previously described.^67^ Hippocampal and iPSC-derived neurons were immunostained with an antibody against MAP2. To analyze the soma size, the area was measured by manually delineating the soma margins. Dendrites were defined as MAP2-positive neurites, and their total lengths were measured. Soma sizes and dendrites were traced and measured manually using freehand tools using ImageJ software (National Institutes of Health, Bethesda, MD, USA).

#### Quantification of mTOR signaling activity and synaptic density

After immunocytochemistry or immunohistochemistry, all images were acquired under identical conditions and analyzed using ImageJ software. *In vitro*, mTOR signaling activity was evaluated by calculating the percentage of p-S6–positive cells relative to Hoechst 33258–positive cells (p-S6/Hoechst [%]), the percentage of p-S6–positive cells relative to S6-positive cells (p-S6/S6 [%]), and the percentage of p-mTOR–positive cells relative to mTOR-positive cells (p-mTOR/mTOR [%]). *In vivo*, brain sections were stained with anti-p-S6 and anti-NeuN antibodies, and the percentage of p-S6–positive neurons relative to NeuN-positive neurons (p-S6/NeuN [%]) was calculated in three to five non-overlapping fields per region (CA3 region of the hippocampus and cerebral cortex) per animal. For synaptic density analysis, brain sections were immunostained with anti-VGLUT1 and anti-PSD95 antibodies. The number of colocalized puncta (overlapping VGLUT1 and PSD95 signals) was counted in six randomly selected 120 μm × 90 μm regions of the hippocampal CA3 per animal. Synaptic density was defined as the total number of colocalized puncta within each fixed-area region.

#### RNA extraction and qRT-PCR

Total RNA from the cells was isolated using the mirVana miRNA Isolation Kit (Ambion, Austin, TX, USA). For detection of mRNAs and primary miRNAs, reverse transcription was performed using the SuperScript VILO cDNA synthesis kit (Invitrogen), followed by qPCR with TaqMan Gene Expression Assays (Applied Biosystems). *GAPDH* was used as the endogenous control. For detection of mature miRNAs, reverse transcription and qPCR were conducted using the TaqMan MicroRNA Reverse Transcription Kit (Applied Biosystems) and TaqMan MicroRNA Assay Kit (Applied Biosystems) according to the manufacturer’s protocol. U6 snRNA was used as the endogenous control for mature miRNAs. All data were analyzed using the comparative Ct method.

#### Two-photon Ca^2+^ imaging of cortical organoids

Two-photon Ca^2+^ imaging was performed using a two-photon fluorescence microscope equipped with a GaAsP-type non-descanned detector and resonant scanner (A1R-MP+, Nikon, Tokyo, Japan) as previously described.^40^ The lower half of each organoid was embedded in 4% low-melting-point agarose and immersed in oxygenated artificial cerebrospinal fluid (ACSF; 125 mM NaCl, 2.5 mM KCl, 1.25 mM NaH_2_PO_4_·H_2_O, 25 mM NaHCO_3_, 25 mM D-glucose, 2 mM CaCl_2_, and 1 mM MgCl_2_). A pulsed laser at 920 nm (InSight DeepSee+, Spectra-Physics, Milpitas, CA, USA) was used to excite jGCaMP7f. Time-lapse images were acquired using a 25× water immersion lens (NA: 1.1, Nikon) at a resolution of 512 × 512 pixels at 30.0 Hz. MATLAB (MathWorks, Natick, MA, USA) was used for image processing and statistical quantification. First, cross-correlation–based rigid image registration was applied to correct for image displacement caused by motion artifacts. Changes in fluorescence intensity in individuamilliporel cells were quantified. The regions of interest in individual cells were semi-manually defined using the “Cell Magic Wand” plugin in ImageJ. To reduce high-frequency artifacts caused by electrical noise, the fluorescence data of jGCaMP7f were smoothed using a Savitzky–Golay filter (filter width = 301 frames). The normalized fluorescence signal change (ΔF/F0) was computed to evaluate the activity of each neuron. The baseline of each cell (F0) was calculated using a low-pass percentile filter (10th percentile; cutoff frequency = 1/15 Hz). Active cells were determined based on three criteria calculated from ΔF/F0: (1) skewness of the ΔF/F0 distribution during recording > 1.0; (2) SD of ΔF/F0 > 0.04; and (3) ≥ 1 detected Ca^2+^ event during the recording period. A Ca^2+^ event in jGCaMP7f was defined as the peak with “MinPeakHeight = 0.2” “MinPeakDistance = 30” and “MinPeakWidth = 15” using the MATLAB findpeaks function. The average amplitude was defined as the mean peak height of all detected Ca^2+^ events during the recording period.

### Quantification and statistical analysis

All quantitative data are presented as mean ± standard deviation (SD). Statistical analyses were performed using GraphPad Prism 10 (GraphPad Software, San Diego, CA, USA). The specific statistical tests used, exact values of n, and what n represents are provided in the corresponding figure legends. Comparisons between two groups were performed using two-tailed Student’s *t*-tests, and multiple group comparisons were analyzed by one-way ANOVA followed by Tukey’s or Dunnett’s multiple comparison tests, as appropriate. Statistical significance was defined as *p* < 0.05.

## References

[1] Chahrour M., Jung S.Y., Shaw C., Zhou X., Wong S.T.C., Qin J., Zoghbi H.Y. (2008). MeCP2, a key contributor to neurological disease, activates and represses transcription. Science.

[2] Nguyen M.V.C., Du F., Felice C.A., Shan X., Nigam A., Mandel G., Robinson J.K., Ballas N. (2012). MeCP2 is critical for maintaining mature neuronal networks and global brain anatomy during late stages of postnatal brain development and in the mature adult brain. J. Neurosci..

[3] Shahbazian M.D., Antalffy B., Armstrong D.L., Zoghbi H.Y. (2002). Insight into Rett syndrome: MeCP2 levels display tissue- and cell-specific differences and correlate with neuronal maturation. Hum. Mol. Genet..

[4] Kishi N., Macklis J.D. (2004). MECP2 is progressively expressed in post-migratory neurons and is involved in neuronal maturation rather than cell fate decisions. Mol. Cell. Neurosci..

[5] Nan X., Ng H.H., Johnson C.A., Laherty C.D., Turner B.M., Eisenman R.N., Bird A. (1998). Transcriptional repression by the methyl-CpG-binding protein MeCP2 involves a histone deacetylase complex. Nature.

[6] Young J.I., Hong E.P., Castle J.C., Crespo-Barreto J., Bowman A.B., Rose M.F., Kang D., Richman R., Johnson J.M., Berget S., Zoghbi H.Y. (2005). Regulation of RNA splicing by the methylation-dependent transcriptional repressor methyl-CpG binding protein 2. Proc. Natl. Acad. Sci. USA.

[7] Cheng T.-L., Wang Z., Liao Q., Zhu Y., Zhou W.-H., Xu W., Qiu Z. (2014). MeCP2 suppresses nuclear microRNA processing and dendritic growth by regulating the DGCR8/Drosha complex. Cell.

[8] Tsujimura K., Irie K., Nakashima H., Egashira Y., Fukao Y., Fujiwara M., Itoh M., Uesaka M., Imamura T., Nakahata Y. (2015). miR-199a Links MeCP2 with mTOR Signaling and Its Dysregulation Leads to Rett Syndrome Phenotypes. Cell Rep..

[9] Amir R.E., Van den Veyver I.B., Wan M., Tran C.Q., Francke U., Zoghbi H.Y. (1999). Rett syndrome is caused by mutations in X-linked MECP2, encoding methyl-CpG-binding protein 2. Nat. Genet..

[10] Chahrour M., Zoghbi H.Y. (2007). The story of Rett syndrome: from clinic to neurobiology. Neuron.

[11] Ananth A., Fu C., Neul J.L., Benke T., Marsh E., Suter B., Ferdinandsen K., Skinner S.A., Annese F., Percy A.K. (2024). MECP2 Variants in Males: More Common than Previously Appreciated. Pediatr. Neurol..

[12] Peters S.U., Fu C., Suter B., Marsh E., Benke T.A., Skinner S.A., Lieberman D.N., Standridge S., Jones M., Beisang A. (2019). Characterizing the phenotypic effect of Xq28 duplication size in MECP2 duplication syndrome. Clin. Genet..

[13] Takeguchi R., Takahashi S., Akaba Y., Tanaka R., Nabatame S., Kurosawa K., Matsuishi T., Itoh M. (2021). Early diagnosis of MECP2 duplication syndrome: Insights from a nationwide survey in Japan. J. Neurol. Sci..

[14] Miguet M., Faivre L., Amiel J., Nizon M., Touraine R., Prieur F., Pasquier L., Lefebvre M., Thevenon J., Dubourg C. (2018). Further delineation of the MECP2 duplication syndrome phenotype in 59 French male patients, with a particular focus on morphological and neurological features. J. Med. Genet..

[15] El Chehadeh S., Touraine R., Prieur F., Reardon W., Bienvenu T., Chantot-Bastaraud S., Doco-Fenzy M., Landais E., Philippe C., Marle N. (2017). Xq28 duplication including MECP2 in six unreported affected females: what can we learn for diagnosis and genetic counselling?. Clin. Genet..

[16] Akaba Y., Takahashi S. (2025). MECP2 duplication syndrome: Recent advances in pathophysiology and therapeutic perspectives. Brain Dev..

[17] Allison K., Maletic-Savatic M., Pehlivan D. (2024). MECP2-related disorders while gene-based therapies are on the horizon. Front. Genet..

[18] Collins A.L., Levenson J.M., Vilaythong A.P., Richman R., Armstrong D.L., Noebels J.L., David Sweatt J., Zoghbi H.Y. (2004). Mild overexpression of MeCP2 causes a progressive neurological disorder in mice. Hum. Mol. Genet..

[19] Sztainberg Y., Chen H.m., Swann J.W., Hao S., Tang B., Wu Z., Tang J., Wan Y.-W., Liu Z., Rigo F. (2015). Reversal of phenotypes in MECP2 duplication mice using genetic rescue or antisense oligonucleotides. Nature.

[20] Bartel D.P. (2018). Metazoan MicroRNAs. Cell.

[21] Kim V.N. (2005). MicroRNA biogenesis: coordinated cropping and dicing. Nat. Rev. Mol. Cell Biol..

[22] Topol A., Zhu S., Hartley B.J., English J., Hauberg M.E., Tran N., Rittenhouse C.A., Simone A., Ruderfer D.M., Johnson J. (2016). Dysregulation of miRNA-9 in a Subset of Schizophrenia Patient-Derived Neural Progenitor Cells. Cell Rep..

[23] Lopez J.P., Lim R., Cruceanu C., Crapper L., Fasano C., Labonte B., Maussion G., Yang J.P., Yerko V., Vigneault E. (2014). miR-1202 is a primate-specific and brain-enriched microRNA involved in major depression and antidepressant treatment. Nat. Med..

[24] Sarachana T., Zhou R., Chen G., Manji H.K., Hu V.W. (2010). Investigation of post-transcriptional gene regulatory networks associated with autism spectrum disorders by microRNA expression profiling of lymphoblastoid cell lines. Genome Med..

[25] Klein M.E., Lioy D.T., Ma L., Impey S., Mandel G., Goodman R.H. (2007). Homeostatic regulation of MeCP2 expression by a CREB-induced microRNA. Nat. Neurosci..

[26] Han K., Gennarino V.A., Lee Y., Pang K., Hashimoto-Torii K., Choufani S., Raju C.S., Oldham M.C., Weksberg R., Rakic P. (2013). Human-specific regulation of MeCP2 levels in fetal brains by microRNA miR-483-5p. Genes Dev..

[27] Nakashima H., Tsujimura K., Irie K., Imamura T., Trujillo C.A., Ishizu M., Uesaka M., Pan M., Noguchi H., Okada K. (2021). MeCP2 controls neural stem cell fate specification through miR-199a-mediated inhibition of BMP-Smad signaling. Cell Rep..

[28] Chen W.G., Chang Q., Lin Y., Meissner A., West A.E., Griffith E.C., Jaenisch R., Greenberg M.E. (2003). Derepression of BDNF transcription involves calcium-dependent phosphorylation of MeCP2. Science.

[29] Vo N., Klein M.E., Varlamova O., Keller D.M., Yamamoto T., Goodman R.H., Impey S. (2005). A cAMP-response element binding protein-induced microRNA regulates neuronal morphogenesis. Proc. Natl. Acad. Sci. USA.

[30] Olson C.O., Pejhan S., Kroft D., Sheikholeslami K., Fuss D., Buist M., Ali Sher A., Del Bigio M.R., Sztainberg Y., Siu V.M. (2018). MECP2 Mutation Interrupts Nucleolin-mTOR-P70S6K Signaling in Rett Syndrome Patients. Front. Genet..

[31] Spruston N. (2008). Pyramidal neurons: dendritic structure and synaptic integration. Nat. Rev. Neurosci..

[32] Ramocki M.B., Peters S.U., Tavyev Y.J., Zhang F., Carvalho C.M.B., Schaaf C.P., Richman R., Fang P., Glaze D.G., Lupski J.R., Zoghbi H.Y. (2009). Autism and other neuropsychiatric symptoms are prevalent in individuals with MeCP2 duplication syndrome. Ann. Neurol..

[33] del Gaudio D., Fang P., Scaglia F., Ward P.A., Craigen W.J., Glaze D.G., Neul J.L., Patel A., Lee J.A., Irons M. (2006). Increased MECP2 gene copy number as the result of genomic duplication in neurodevelopmentally delayed males. Genet. Med..

[34] Lugtenberg D., Kleefstra T., Oudakker A.R., Nillesen W.M., Yntema H.G., Tzschach A., Raynaud M., Rating D., Journel H., Chelly J. (2009). Structural variation in Xq28: MECP2 duplications in 1% of patients with unexplained XLMR and in 2% of male patients with severe encephalopathy. Eur. J. Hum. Genet..

[35] Ebert M.S., Neilson J.R., Sharp P.A. (2007). MicroRNA sponges: competitive inhibitors of small RNAs in mammalian cells. Methods.

[36] Chao H.-T., Zoghbi H.Y., Rosenmund C. (2007). MeCP2 controls excitatory synaptic strength by regulating glutamatergic synapse number. Neuron.

[37] Kim H.W., Ha S.H., Lee M.N., Huston E., Kim D.-H., Jang S.K., Suh P.-G., Houslay M.D., Ryu S.H. (2010). Cyclic AMP controls mTOR through regulation of the dynamic interaction between Rheb and phosphodiesterase 4D. Mol. Cell Biol..

[38] Irie K., Tsujimura K., Nakashima H., Nakashima K. (2016). MicroRNA-214 Promotes Dendritic Development by Targeting the Schizophrenia-associated Gene Quaking (Qki). J. Biol. Chem..

[39] Irie K., Nakashima H., Yamaguchi M., Osakada F., Ozaki N., Tsujimura K., Nakashima K. (2025). MeCP2 controls dendritic morphogenesis via miR-199a-mediated Qki downregulation. bioRxiv.

[40] Nishimura M., Kodera T., Adachi S., Sato A.Y., Takeuchi R.F., Nonaka H., Hamachi I., Osakada F. (2025). Conversion of silent synapses to AMPA receptor-mediated functional synapses in human cortical organoids. Neurosci. Res..

[41] Varghese M., Keshav N., Jacot-Descombes S., Warda T., Wicinski B., Dickstein D.L., Harony-Nicolas H., De Rubeis S., Drapeau E., Buxbaum J.D. (2017). Autism spectrum disorder: neuropathology and animal models. Acta Neuropathol..

[42] Xu X., Miller E.C., Pozzo-Miller L. (2014). Dendritic spine dysgenesis in Rett syndrome. Front. Neuroanat..

[43] Jiang M., Ash R.T., Baker S.A., Suter B., Ferguson A., Park J., Rudy J., Torsky S.P., Chao H.-T., Zoghbi H.Y. (2013). Dendritic arborization and spine dynamics are abnormal in the mouse model of MECP2 duplication syndrome. J. Neurosci..

[44] Isshiki M., Tanaka S., Kuriu T., Tabuchi K., Takumi T., Okabe S. (2014). Enhanced synapse remodelling as a common phenotype in mouse models of autism. Nat. Commun..

[45] Bodda C., Tantra M., Mollajew R., Arunachalam J.P., Laccone F.A., Can K., Rosenberger A., Mironov S.L., Ehrenreich H., Mannan A.U. (2013). Mild overexpression of Mecp2 in mice causes a higher susceptibility toward seizures. Am. J. Pathol..

[46] Bajikar S.S., Sztainberg Y., Trostle A.J., Tirumala H.P., Wan Y.-W., Harrop C.L., Bengtsson J.D., Carvalho C.M.B., Pehlivan D., Suter B. (2024). Modeling antisense oligonucleotide therapy in MECP2 duplication syndrome human iPSC-derived neurons reveals gene expression programs responsive to MeCP2 levels. Hum. Mol. Genet..

[47] Takei N., Nawa H. (2014). mTOR signaling and its roles in normal and abnormal brain development. Front. Mol. Neurosci..

[48] Lipton J.O., Sahin M. (2014). The neurology of mTOR. Neuron.

[49] Butler M.G., Dasouki M.J., Zhou X.-P., Talebizadeh Z., Brown M., Takahashi T.N., Miles J.H., Wang C.H., Stratton R., Pilarski R., Eng C. (2005). Subset of individuals with autism spectrum disorders and extreme macrocephaly associated with germline PTEN tumour suppressor gene mutations. J. Med. Genet..

[50] Hobert J.A., Embacher R., Mester J.L., Frazier T.W., Eng C. (2014). Biochemical screening and PTEN mutation analysis in individuals with autism spectrum disorders and macrocephaly. Eur. J. Hum. Genet..

[51] Kwon C.-H., Luikart B.W., Powell C.M., Zhou J., Matheny S.A., Zhang W., Li Y., Baker S.J., Parada L.F. (2006). Pten regulates neuronal arborization and social interaction in mice. Neuron.

[52] Zhou J., Blundell J., Ogawa S., Kwon C.-H., Zhang W., Sinton C., Powell C.M., Parada L.F. (2009). Pharmacological inhibition of mTORC1 suppresses anatomical, cellular, and behavioral abnormalities in neural-specific Pten knock-out mice. J. Neurosci..

[53] Buist M., El Tobgy N., Shevkoplyas D., Genung M., Sher A.A., Pejhan S., Rastegar M. (2022). Differential Sensitivity of the Protein Translation Initiation Machinery and mTOR Signaling to MECP2 Gain- and Loss-of-Function Involves MeCP2 Isoform-Specific Homeostasis in the Brain. Cells.

[54] Lin E.A., Kong L., Bai X.-H., Luan Y., Liu C.-J. (2009). miR-199a, a bone morphogenic protein 2-responsive MicroRNA, regulates chondrogenesis via direct targeting to Smad1. J. Biol. Chem..

[55] Nakashima H., Tsujimura K., Irie K., Ishizu M., Pan M., Kameda T., Nakashima K. (2018). Canonical TGF-β Signaling Negatively Regulates Neuronal Morphogenesis through TGIF/Smad Complex-Mediated CRMP2 Suppression. J. Neurosci..

[56] Nageshappa S., Carromeu C., Trujillo C.A., Mesci P., Espuny-Camacho I., Pasciuto E., Vanderhaeghen P., Verfaillie C.M., Raitano S., Kumar A. (2016). Altered neuronal network and rescue in a human MECP2 duplication model. Psychiatry.

[57] Swann J.W., Al-Noori S., Jiang M., Lee C.L. (2000). Spine loss and other dendritic abnormalities in epilepsy. Hippocampus.

[58] Ta D., Downs J., Baynam G., Wilson A., Richmond P., Leonard H. (2022). A brief history of MECP2 duplication syndrome: 20-years of clinical understanding. Orphanet J. Rare Dis..

[59] Montgomery K.R., Louis Sam Titus A.S.C., Wang L., D’Mello S.R. (2018). Elevated MeCP2 in Mice Causes Neurodegeneration Involving Tau Dysregulation and Excitotoxicity: Implications for the Understanding and Treatment of MeCP2 Triplication Syndrome. Mol. Neurobiol..

[60] Aldinger K.A., Timms A.E., MacDonald J.W., McNamara H.K., Herstein J.S., Bammler T.K., Evgrafov O.V., Knowles J.A., Levitt P. (2020). Transcriptome data of temporal and cingulate cortex in the Rett syndrome brain. Data.

[61] Mietto M., Montanari S., Falzarano M.S., Manzati E., Rimessi P., Fabris M., Selvatici R., Gualandi F., Neri M., Fortunato F. (2025). MECP2 mRNA Profile in Brain Tissues from a Rett Syndrome Patient and Three Human Controls: Mutated Allele Preferential Transcription and In Situ RNA Mapping. Biomolecules.

[62] Colantuoni C., Jeon O.H., Hyder K., Chenchik A., Khimani A.H., Narayanan V., Hoffman E.P., Kaufmann W.E., Naidu S., Pevsner J. (2001). Gene expression profiling in postmortem Rett Syndrome brain: differential gene expression and patient classification. Neurobiol. Dis..

[63] Gioiosa S., Gasparini S., Presutti C., Rinaldi A., Castrignanò T., Mannironi C. (2025). Integrated gene expression and alternative splicing analysis in human and mouse models of Rett syndrome. Sci. Rep..

[64] Arioka Y., Shishido E., Kushima I., Suzuki T., Saito R., Aiba A., Mori D., Ozaki N. (2021). Chromosome 22q11.2 deletion causes PERK-dependent vulnerability in dopaminergic neurons. EBioMedicine.

[65] Takahashi K., Tanabe K., Ohnuki M., Narita M., Ichisaka T., Tomoda K., Yamanaka S. (2007). Induction of pluripotent stem cells from adult human fibroblasts by defined factors. Cell.

[66] Tsujimoto H., Katagiri N., Ijiri Y., Sasaki B., Kobayashi Y., Mima A., Ryosaka M., Furuyama K., Kawaguchi Y., Osafune K. (2022). In vitro methods to ensure absence of residual undifferentiated human induced pluripotent stem cells intermingled in induced nephron progenitor cells. PLoS One.

[67] Akaba Y., Takahashi S., Suzuki K., Kosaki K., Tsujimura K. (2023). miR-514a promotes neuronal development in human iPSC-derived neurons. Front. Cell Dev. Biol..

[68] Kaech S., Banker G. (2006). Culturing hippocampal neurons. Nat. Protoc..

[69] Arioka Y., Shishido E., Kubo H., Kushima I., Yoshimi A., Kimura H., Ishizuka K., Aleksic B., Maeda T., Ishikawa M. (2018). Single-cell trajectory analysis of human homogenous neurons carrying a rare RELN variant. Psychiatry.

[70] Suzuki T., Morimoto N., Akaike A., Osakada F. (2019). Multiplex Neural Circuit Tracing With G-Deleted Rabies Viral Vectors. Circuits.

