# Supplementary material for: miR-199a functions downstream of MeCP2 in neurons of MECP2 duplication syndrome models
Source: iScience. 2025 Oct 16;28(11):113789. doi: 10.1016/j.isci.2025.113789 (PMC12613072; doi:10.1016/j.isci.2025.113789)
Supplement: Document S1. Figures S1 and S2, Table S1, and Data S1 [file mmc1.pdf]

iScience, Volume *28*

Supplemental information

miR-199a functions downstream of MeCP2 in neurons of *MECP2* duplication syndrome models

Yuichi Akaba, Satoru Takahashi, Shota Adachi, Masatoshi Nishimura, Keiichiro Suzuki, Hideyuki Nakashima, Kinichi Nakashima, Ryutaro Kira, Pin Fee Chong, Yasunari Sakai, Yohei Hayashi, Itaru Kushima, Daisuke Mori, Yuko Arioka, Hiroki Okumura, Atsuo Nakayama, Seiji Mizuno, Toshiyuki Yamamoto, Fumitaka Osakada, Norio Ozaki, and Keita Tsujimura

Fig. S1

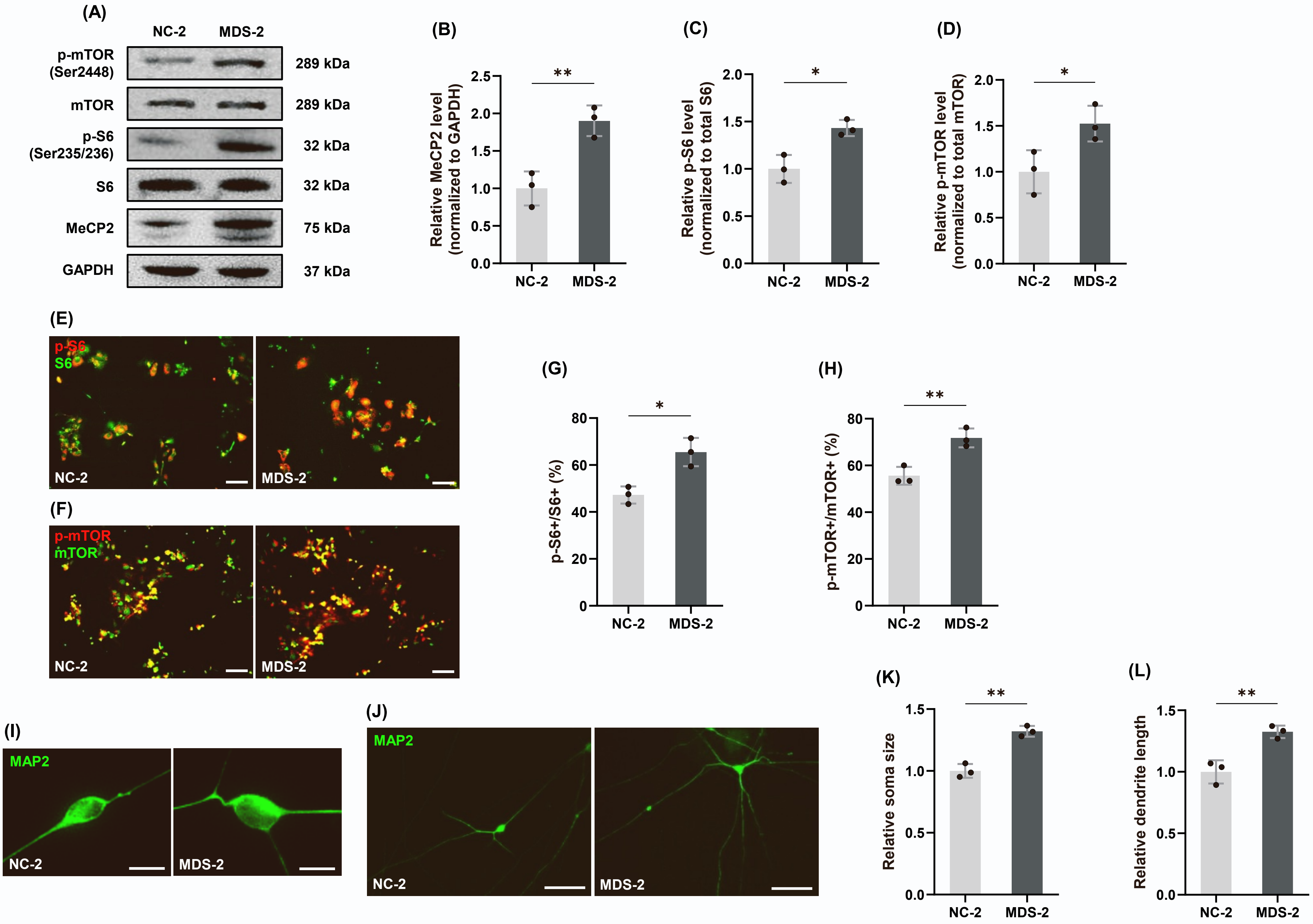
(A)
NC-2
MDS-2
p-mTOR (Ser2448)
mTOR
p-S6 (Ser235/236)
S6
MeCP2
GAPDH
289 kDa
289 kDa
32 kDa
32 kDa
75 kDa
37 kDa
(B)
Relative MeCP2 level (normalized to GAPDH)
**
(C)
Relative p-S6 level (normalized to total S6)
*
(D)
Relative p-mTOR level (normalized to total mTOR)
*
(E)
p-S6
S6
NC-2
MDS-2
(F)
p-mTOR
mTOR
NC-2
MDS-2
(G)
p-S6+/S6+ (%)
*
(H)
p-mTOR+/mTOR+ (%)
**
(I)
MAP2
NC-2
MDS-2
(J)
MAP2
NC-2
MDS-2
(K)
Relative soma size
**
(L)
Relative dendrite length
**

Fig. S1. Dysregulated mTOR signaling and morphological abnormalities in iPSC-derived MDS neurons.

(A) Representative Western blot images of MeCP2, p-S6, total S6, p-mTOR, total mTOR, and GAPDH in human iPSC-derived neurons at 7 DIV. Samples from NC-2 and MDS-2 were lysed for immunoblotting. The MeCP2/GAPDH ratio was used to evaluate MeCP2 expression levels, while the p-S6/S6 and p-mTOR/mTOR ratios were used to assess mTOR signaling activity. GAPDH was used as a loading control.

(B, C, D) Quantification of protein expression levels shown in (A) using iPSC-derived neurons from two lines: NC-2 and MDS-2. Neurons were lysed at 7 DIV for Western blot analysis. Data are presented as mean ± SD from three independent experiments (n = 3). The average relative MeCP2/GAPDH ratio (B) was 1.00 ± 0.23 in NC-2 and 1.90 ± 0.20 in MDS-2. The average relative p-S6/S6 ratio (C) was 1.00 ± 0.15 in NC-2 and 1.43 ± 0.09 in MDS-2. The average relative p-mTOR/mTOR ratio (D) was 1.00 ± 0.24 in NC-2 and 1.52 ± 0.19 in MDS-2. Statistical significance was determined using Student's *t*-test (*$p < 0.05$; **$p < 0.01$).

(E, F) Representative images of human iPSC-derived neurons at 7 DIV stained with anti-p-S6 (red) and anti-S6 (green) antibodies (E), or with anti-p-mTOR (red) and anti–mTOR (green) antibodies (F). Scale bars: 100 μm.

(G, H) Quantification of mTOR activity at 7 DIV using human iPSC-derived neurons from two lines: NC-2 and MDS-2. Data are presented as mean ± SD from three independent experiments (n = 3). In all experiments, at least 53 neurons were analyzed per condition. p-S6-positive cells accounted for 47.2% ± 3.7% of S6-positive cells in NC-2 and 65.5% ± 6.0% in MDS-2 (G), and p-mTOR-positive cells accounted for 55.6% ± 3.8% of mTOR-positive cells in NC-2 and 71.8% ± 4.1% in MDS-2 (H). Statistical significance was determined using Student's *t*-test (*$p < 0.05$; **$p < 0.01$).

(I, J) Representative images of human iPSC-derived neurons stained with anti-MAP2 (green) antibody at 14 DIV. Scale bars: 20 μm (I), 70 μm (J).

(K, L) Quantification of neuronal soma size (K) and total dendrite length (L) at 14 DIV using human iPSC-derived neurons from NC-2 and MDS-2. Data are presented as mean ± SD from three independent experiments (n = 3). In all experiments, at least 50 neurons were analyzed per condition. The average relative soma size was 1.00 ± 0.06 in NC-2 and 1.32 ± 0.04 in MDS-2. The average relative dendrite length was 1.00 ± 0.09 in NC-2 and 1.33 ± 0.05 in MDS-2. Statistical significance was determined using Student's *t*-test (***p*

< 0.01).

Fig. S2

(A)

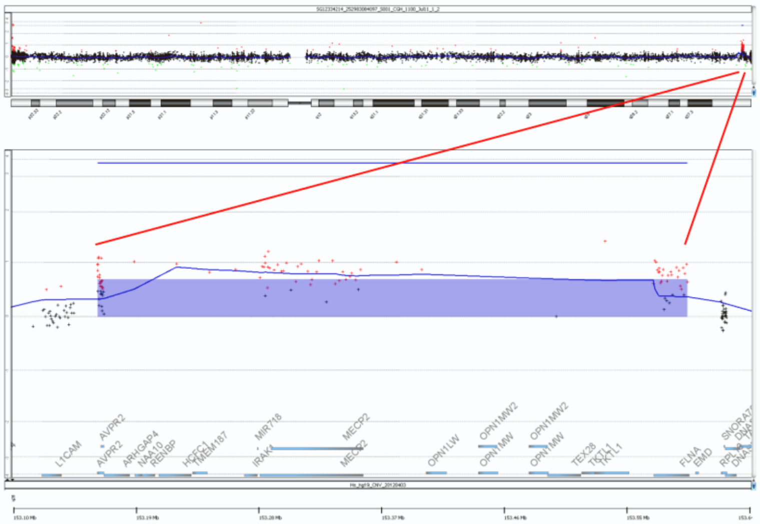

Genes encompassed in the duplication region at Xq28: *AVPR2*, *ARHGAP4*, *NAA10*, *RENBP*, *HCFC1*, *TMEM187*, *IRAK1*, *MIR718*, *MECP2*, *OPN1LW*, *OPN1MW*, *OPN1MW2*, *TEX28*, *TKTL1*, and *FLNA* genes

(B)

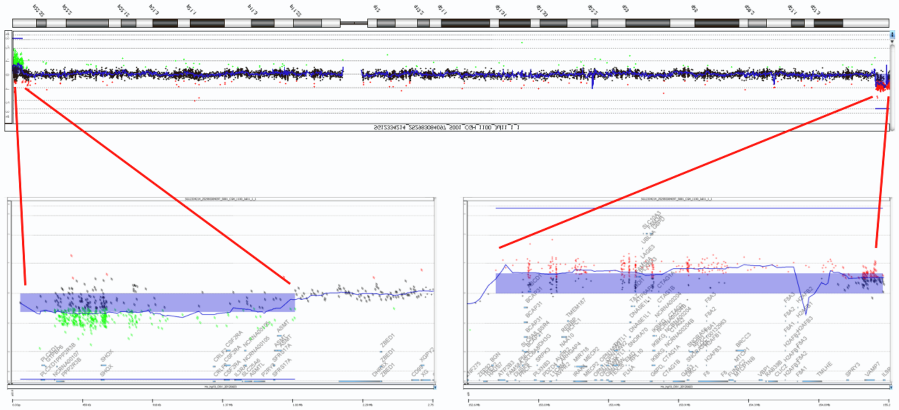

Genes encompassed in the deletion region at Xp22.33: *PLCXD1*, *GTPBP6*, *NCRNA00107*, *PPP2R3B*, *SHOX*, *CRLF2*, *CSF2RA*, *IL3RA*, *SLC25A6*, *NCRNA00105*, *ASMTL*, *P2RY8*, *SFRS17A*, and *ASMT* genes

Genes encompassed in the duplication region at Xq28: *ATP2B3*, *FAM58A*, *DUSP9*, *PNCK*, *SLC6A8*, *BCAP31*, *ABCD1*, *PLXNB3*, *SRPK3*, *IDH3G*, *SSR4*, *PDZD4*, *L1CAM*, *AVPR2*, *ARHGAP4*, *NAA10*, *RENBP*, *HCFC1*, *TMEM187*, *IRAK1*, *MIR718*, *MECP2*, *OPN1LW*, *OPN1MW*, *OPN1MW2*, *TEX28*, *TKTL1*, *FLNA*, *EMD*, *RPL10*, *SNORA70*, *DNASE1L1*, *TAZ*, *ATP6AP1*, *GDI1*, *FAM50A*, *PLXNA3*, *LAGE3*, *UBL4A*, *SLC10A3*, *FAM3A*, *G6PD*, *IKBKG*, *NCRNA00204B*, *NCRNA00204*, *CTAG1B*, *CTAG1A*, *CTAG2*, *GAB3*, *DKC1*, *SNORA36A*, *SNORA56*, *MPP1*, *LOC100132963*, *F8*, *H2AFB3*, *H2AFB1*, *F8A1*, *F8A2*, *F8A3*, *FUNDC2*, *MTCP1NB*, *MTCP1*, *BRCC3*, *VBP1*, *RAB39B*, *CLIC2*, *H2AFB2*, *TMLHE*, *SPRY3*, and *VAMP7* genes

(C)

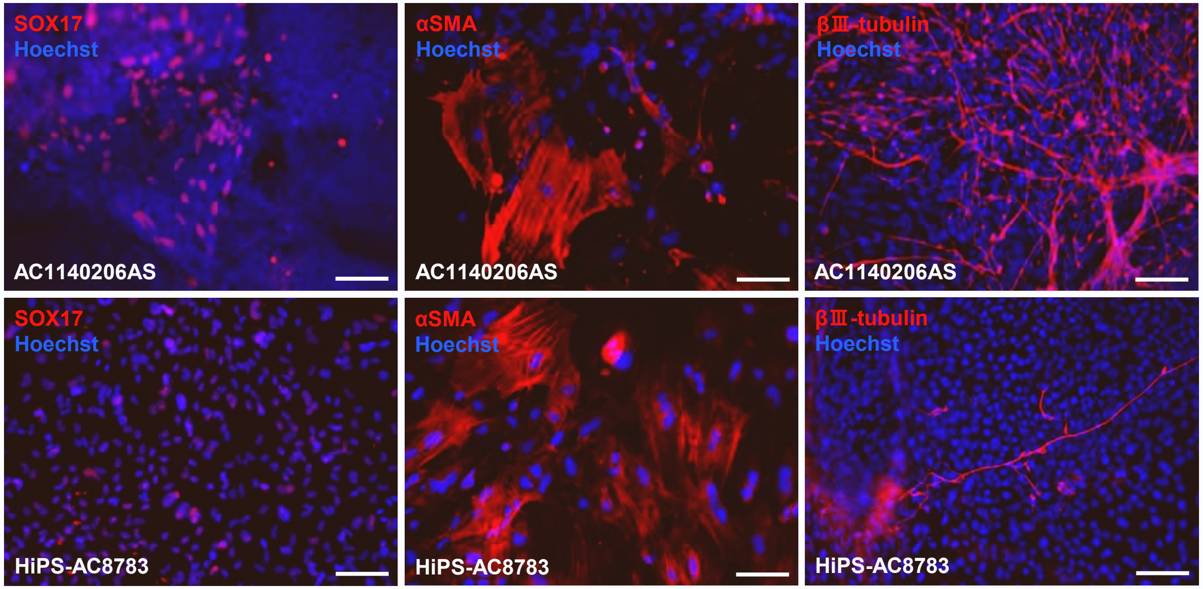

Fig. S2. Genomic characterization and pluripotency validation of iPSCs derived from MDS patients.

(A) CGH analysis of patient 1 shows an ~434 kb duplication at Xq28 encompassing 15 genes including *MECP2*.

(B) CGH analysis of patient 2 shows an ~1.8 Mb deletion at Xp22.33 encompassing 14 genes and an ~2.4 Mb duplication at Xq28 encompassing 71 genes including *MECP2*.

(C) *In vitro* three-germ-layer differentiation assay by forming embryoid bodies (EBs). For EB formation, 1.2 x 106 cells were suspended in StemFit AK02N (Ajinomoto, Tokyo, Japan) into the V-shape 96-well plate. From day 2, these cell aggregates were cultured in DMEM high glucose (Nacalai) supplemented with 10% fetal bovine serum (Biosera, Cholet, France) (EB medium) for 8 days and plated on 0.1(w/v)% gelatin solution (Wako)-coated plate in the EB medium for another 8 days. The capacity of iPSCs to differentiate into all three germ layers was evaluated using SOX17 staining (endodermal marker), αSMA staining (mesodermal marker), and βIII-tubulin staining (ectodermal marker). Scale bars: 100 µm.

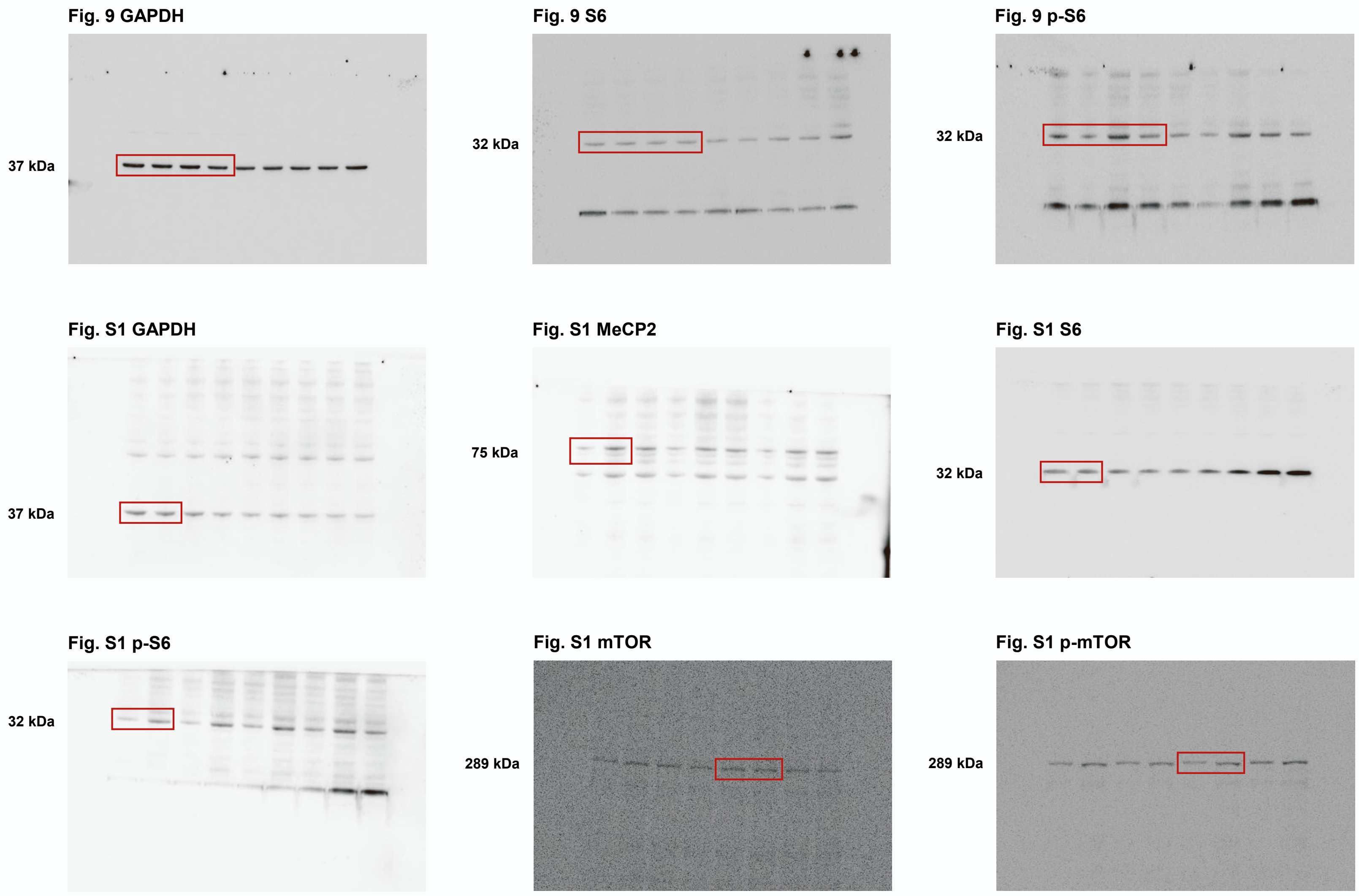

Data S1. Raw, uncropped images of the immunoblot membranes presented in Figures 9 and S1.

Table S1. Clinical features of two patients with MDS

	Patient 1	Patient 2
Gender	male	male
Age at the time of the study	13y	14y
Family history	familial*	sporadic
Dysmorphic features	+	+
Hypotonia	+	+
Recurrent infections	+	+
Gross motor development	delayed	delayed
Head control	4mo	4mo
Sitting alone	10mo	9mo
Walking alone	2y6mo	1y6mo
Intellectual disability	severe	severe
Autistic features	+	+
Stereotypic movements	−	+
Seizures (age at onset)	−	+ (6y)
Sleep problems	−	+
Bruxism	+	−
Constipation	−	+
Gastroesophageal reflex	+	+

*The elder brother was diagnosed with genetically confirmed MDS.

Abbreviations: MDS, *MECP2* duplication syndrome; y, years; mo, months
